# Supplementary material for: Snowfall decrease in recent years undermines glacier health and meltwater resources in the Northwestern Pamirs
Source: Commun Earth Environ. 2025 Sep 2;6(1):691. doi: 10.1038/s43247-025-02611-8 (PMC12404989; doi:10.1038/s43247-025-02611-8)
Supplement: Supplementary file 2 — Supplementary Information [file 43247_2025_2611_MOESM2_ESM.pdf]

Supplementary material to: **Snowfall decrease in recent years undermines glacier health and meltwater resources in the Northwestern Pamirs**

Achille Jouberton[1,2,3], Thomas E. Shaw[1], Evan Miles[3,4,5], Marin Kneib[6,7], Stefan Fugger[1,2,3], Pascal Buri[1,3,8], Michael McCarthy[1,3], Abdulhamid Kayumov[9], Hofiz Navruzshoev[9], Ardamehr Halimov[9], Khusrav Kabutov[9], Farrukh Homidov [9], Francesca Pellicciotti[1]

1. Institute of Science and Technology Austria, ISTA, Klosterneuburg, Austria
2. Institute of Environmental Engineering, ETH Zurich, Zurich, Switzerland
3. Swiss Federal Research Institute WSL, Birmensdorf, Switzerland
4. University of Zurich, Department of Geography, Glaciology and Geomorphodynamics Group, Zürich, Switzerland
5. Department of Geosciences, University of Fribourg, Switzerland
6. Institut des Géosciences de l'Environnement, Université Grenoble-Alpes, CNRS, IRD, Grenoble, France
7. Department of Atmospheric and Cryospheric Sciences, University of Innsbruck, Innsbruck, Austria
8. Geophysical Institute, University of Alaska Fairbanks, Fairbanks, USA
9. Center for the Research of Glaciers of the Tajik Academy of Tajikistan, Dushanbe, Tajikistan
10. Mountain Societies Research Institute, University of Central Asia, Dushanbe 734000, Tajikistan

Table of contents:

1. Supplementary methods: Hydrometeorological observations........ 3
2. Supplementary methods: downscaling of meteorological forcing........ 6
3. Supplementary methods: remote sensing of glacier and snow changes........ 11
 3.1 High-resolution snow cover mapping........ 11
 3.2 Spatially resolved snow depth from very high-resolution stereo imagery........ 15
4. Supplementary methods: Model set-up........ 17
 4.1 Land covers........ 17
 4.2 Initial conditions........ 19
 4.3 Glacier dynamics........ 20
 4.4 Gravitational redistribution........ 22
 4.5 Model limitation: lack of firn representation........ 24
5. Supplementary methods: model evaluation........ 25
 5.1 Point-scale evaluation........ 26
 5.2 Distributed scale evaluation........ 30
6. Supplementary Methods: Cut-off year for sub-periods comparison........ 35
7. Supplementary Note 1: Seasonal and altitudinal additional model outputs........ 37
8. Supplementary Note 2: Linear regression analyses........ 39
9. Supplementary Note 3: Additional material........ 41

10. References.. 42

List of tables and figures:

Table S1. Coordinates and variables recorded by meteorological stations
Table S2. Summary of model performance metrics, evaluated against observations

Figure S1. Photos of hydrometeorological stations used in this study
Figure S2. Measured air temperature lapse rates
Figure S3. Precipitation undercatch correction and snow depth simulation
Figure S4. ERA5-Land downscaling evaluated at Pluviometer station
Figure S5. ERA5-Land downscaling evaluated at AWS on-glacier
Figure S6. ERA5-Land downscaling evaluated at AWS ridge
Figure S7. ERA5-Land downscaling evaluated at distributed temperature loggers
Figure S8. Seasonality of ERA5-Land precipitation before and after bias correction
Figure S9: Spatial variability of ERA5-Land precipitation at Kyzylsu catchment
Figure S10: Classification of clean-ice areas for snow cover mapping
Figure S11: Snow cover mapping example for an end of summer Sentinel-2 scene
Figure S12: Snow cover mapping example for an October scene
Figure S13: Ortho-images of Pléiades scenes used in this study
Figure S14: Co-registration and elevation change derived from Pleiades DEM differencing
Figure S15: Spatial model setup of land covers
Figure S16: Spatial model setup of glacier and debris cover areas
Figure S17: Remotely sensed glacier albedo per elevation bands
Figure S18: Model initial conditions of snow cover and snow depth
Figure S19: Glacier outlines comparison between 2000 and 2022
Figure S20: Elevation change over 2000-2019 from Hugonnet et al. 2021
Figure S21: Evaluation of simulated avalanche deposits against Pléiades elevation change
Figure S22: Evaluation of simulated avalanche deposits against Sentinel-1 data
Figure S23: The sensitivity of glacier mass balance to the representation of avalanches
Figure S24: Evaluation of simulated snow albedo against in-situ observation
Figure S25: Evaluation of simulated glacier mass balance against ablation stakes
Figure S26: Evaluation of simulated surface elevation change at AWS on-glacier
Figure S27: Evaluation of simulated snow depth against in-situ observation
Figure S28: Evaluation of simulated water level at the proglacial stream of Koshkul Glacier.
Figure S29: Evaluation of modelled catchment fractional snow cover (2000-2011)
Figure S30: Evaluation of modelled catchment fractional snow cover (2012-2023)
Figure S31: Evaluation of modelled snow line elevation (2000-2011)
Figure S32: Evaluation of modelled snow line elevation (2012-2023)
Figure S33: Snowpack simulations at Pluviometer for all hydrological years
Figure S34: Breakpoint analysis of annual precipitation, temperature, glacier mass balance
Figure S35: Regional anomalies in precipitation and snow cover between 2000-2012 and 2012-2023
Figure S36: Mean seasonal air temperature averaged over the Kyzylsu catchment since 1999
Figure S37: Mean seasonal snowfall fraction per 100-m elevation bands
Figure S38: Mean seasonal snowfall and precipitation per 100-m elevation bands

Figure S39: Mean monthly elevation of the 0°C isotherm for all hydrological years
Figure S40: Mean annual evapotranspiration simulated over the Kyzylsu catchment
Figure S41: Linear regression between snowpack melt-out and meteorological conditions.
Figure S42: Linear regression between annual snowfall, mass balance and temperature
Figure S43: Linear regression between annual snowfall, precipitation and temperature
Figure S44: Daily maximum air temperature recorded in 2022 at the Dushanbe Airport.
Figure S45: Changes in energy fluxes and surface albedo of Kyzylsu Glacier

1. Supplementary methods 1: Hydrometeorological observations

Table S1: Coordinates and variables recorded of the main hydrometeorological stations installed at Kyzylsu catchment. Ta: air temperature, RH: relative humidity, WS: wind speed, WD: wind direction, Swin/out: incoming and outgoing shortwave radiation, Lwin/out: incoming and outgoing longwave radiation, SND: snow depth.

**Station**	**Lat [°]**	**Lon [°]**	**Elevation [m]**	**Variable recorded**	**Observation period:**
AWS on-glacier	39.09697	71.41769	3579	Ta, RH, WS, WD, Swin/out, LWin/out, HS	July 2021 - Sep 2023
Pluviometer station	39.11507	71.41184	3369	P, Ta, RH, WS, WD, Swin/out, SND	July 2021 - Sep 2023
AWS ridge	39.10005	71.43203	3910	Ta, WD, WS, RH, Swin/out, SND	Sep 2022 - Sep 2023
Muk outlet	39.1578	71.54683	2130	Ta	Jun 2022 - Sep 2022
Koshkul stream	39.1343	71.45033	3157	Stream water level	July 2021 - Sep 2023
Alpine village	39.13514	71.4448	3201	Ta, SND	Jul 2021 - Sep 2023

Ridge 3500m	39.10609	71.42113	3505	Ta	Jul 2021 - Sep 2023
Ridge 3900m	39.10005	71.43203	3910	Ta	Jul 2021 - Sep 2022
On-glacier 3695m	39.08273	71.42043	3695	SND	Sep 2022 - Sep 2023

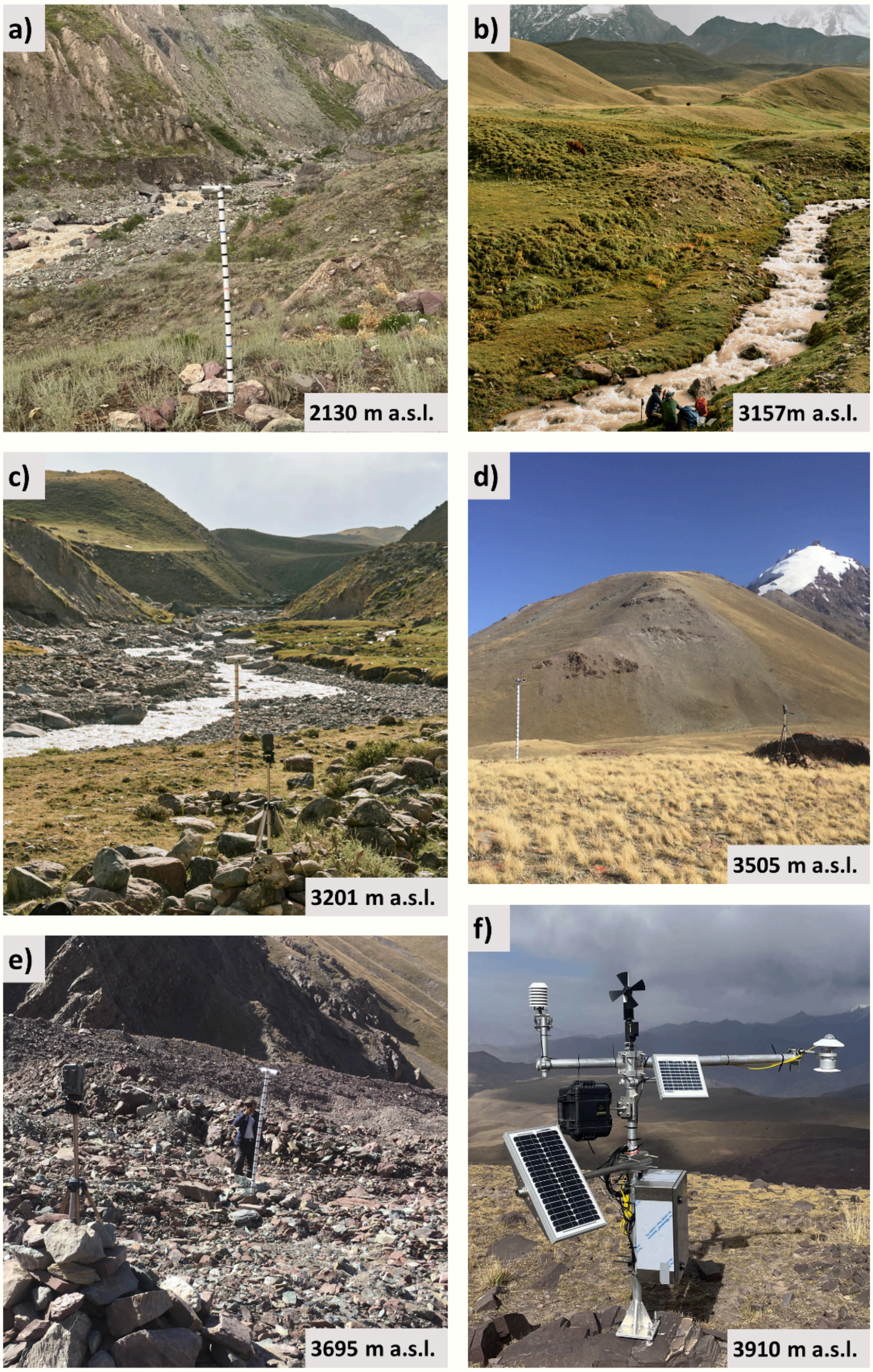

Figure S1: Photos of hydrometeorological stations used in this study, in addition to the pluviometer station and on-glacier AWS displayed in Figure 1. a) Air temperature logger located at the catchment outlet, near the village of Muk. b) Koshkul proglacial stream where a HOBO level logger was attached to an aluminum bar submerged in the water. c) Air temperature logger with graduated mast and time-lapse camera used to retrieve daily snow depth, located near the Alpine village. d) Air temperature logger located on a ridge near Kyzylsu Glacier, at 3505m a.s.l.. e) Air temperature logger with graduated mast and time-lapse camera used to retrieve daily snow depth, located on the debris-covered portion of Kyzylsu Glacier. (f) Ridge AWS.

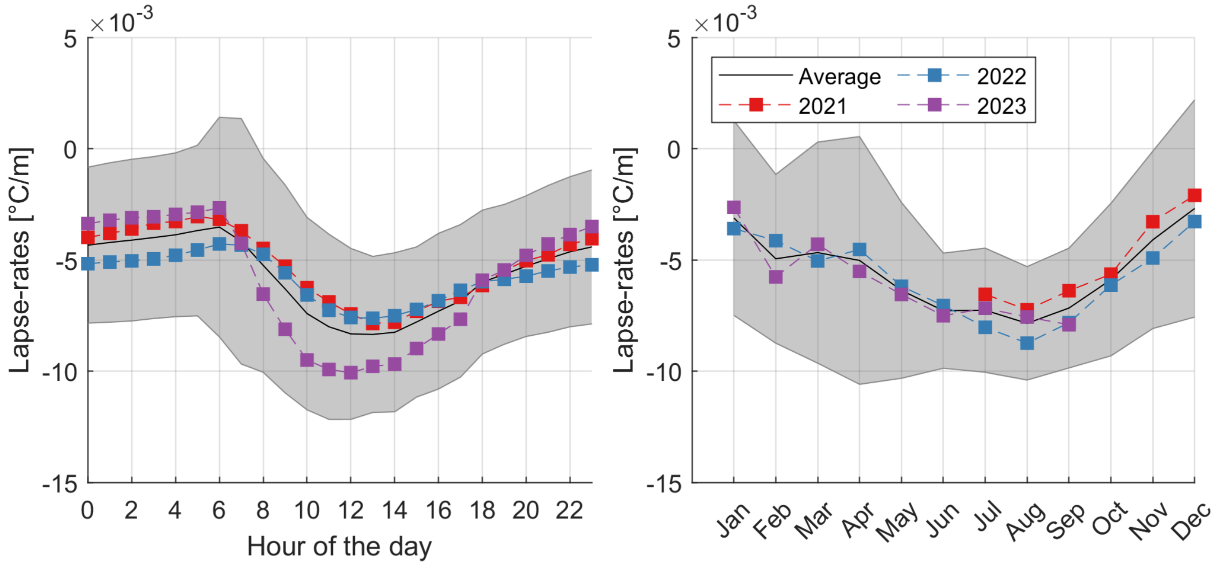

Figure S2. Air temperature lapse rates derived from simultaneous hourly measurements of air temperature at different elevations in the Kyzylsu catchment. All T-logger stations used to derive these lapse rates were located on off-glacier terrains. (left) Mean air temperature lapse rates per hour of the day averaged over the Jul 2021 - 2023 period. (right) Mean air temperature lapse rates per month of the year averaged over the Jul 2021 - 2023 period.

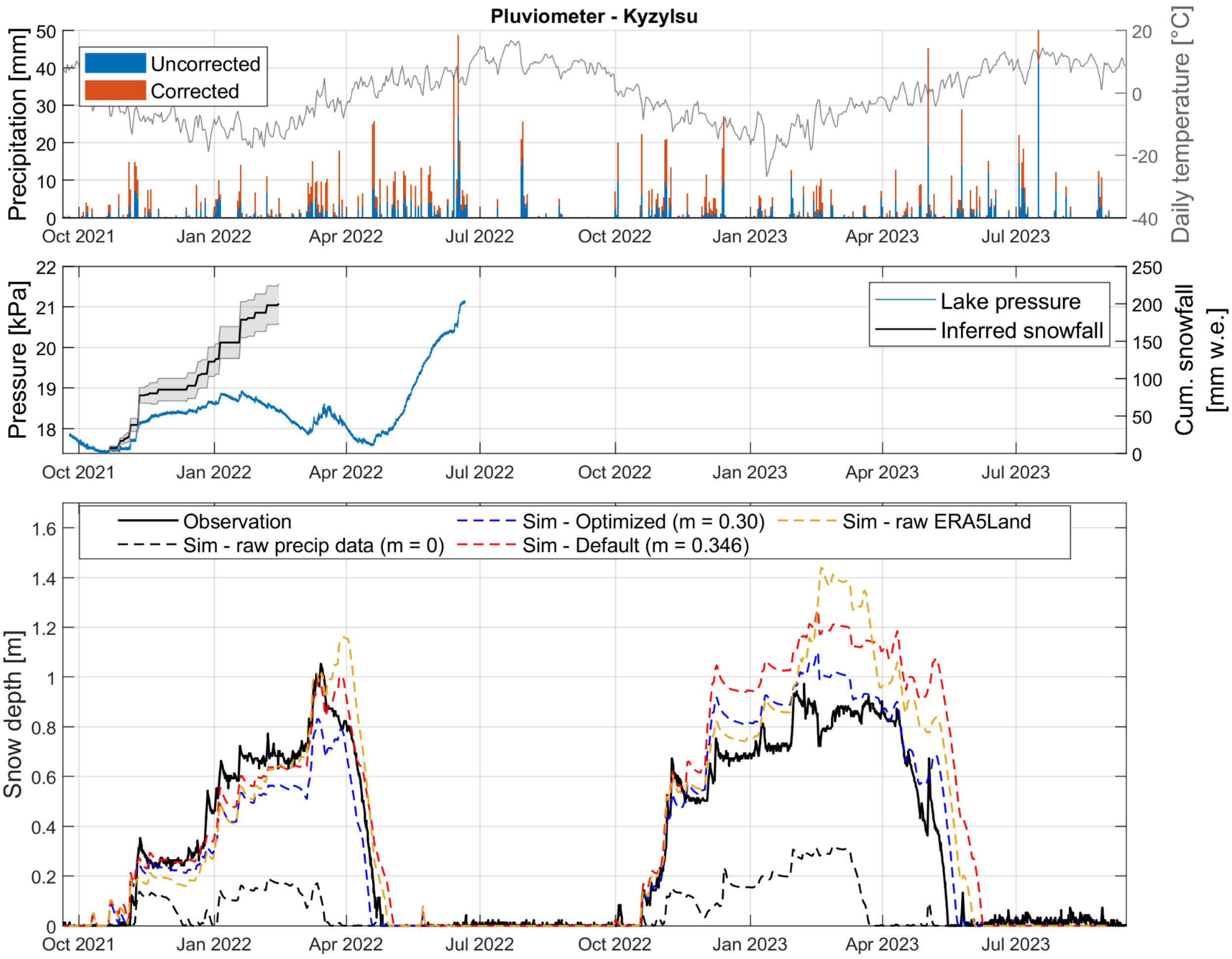

Figure S3. Meteorological and snow conditions at the pluviometer station. (top) Measured mean daily air temperature and daily precipitation, with and without undercatch correction. (middle) Pressure measured at the bottom of Maidakul Lake, after subtracting the atmospheric pressure recorded by a similar logger located outside of the water in the lake's proximity. The cumulative snowfall inferred from the pressure signal is shown in black, with the shaded grey area corresponding to the snowfall uncertainty as quantified in Pritchard et al. 2021. (bottom) Daily snow depth observed using an ultrasonic depth gauge (solid black line), compared to our snowpack height simulations conducted for several precipitation undercatch correction factors (m) or using the raw (non-bias corrected) ERA5-Land precipitation. For this set of point-scale simulations, we used measured air temperature, wind speed, relative humidity and bias-corrected ERA5-Land pressure, incoming shortwave and longwave radiations.

2. Supplementary methods 2: downscaling of meteorological forcing

The bias correction of precipitation was conducted independently for the twelve months of the year, on daily sums of precipitation measured using the undercatch corrected precipitation record displayed in Fig. S3. From 20 September 2021 to 12 September 2023, 712 mm of precipitation was measured at the pluviometer station location (3369 m a.s.l.), corresponding to 1605 mm after the undercatch correction using the optimized (default) solid

precipitation correction coefficient. For the same period, the ERA5-Land downscaled and bias-corrected precipitation amounts to 1606 mm, nearly identical to the corrected measured amounts. The non-bias corrected ERA5-Land precipitation would be 1564 mm, also very close to the measured amounts, with notable differences in seasonality. Relative to the measurement, ERA5-Land gives higher precipitation amounts in January to March, but less in May to July.

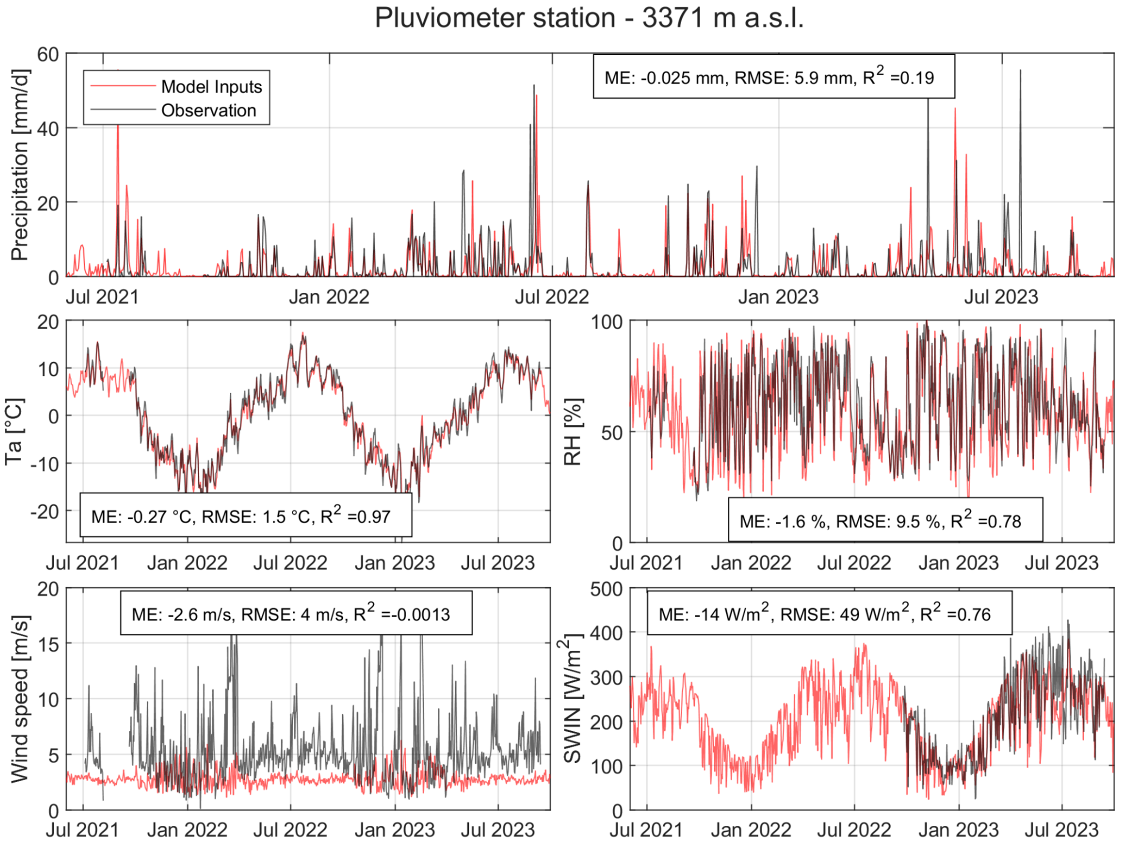

Figure S4. Evaluation of ERA5-Land downscaled and bias-corrected meteorological forcing at the location of the pluviometer location which has been operating since June 2021. All variables are shown at hourly time scales except for precipitation. (a) Daily precipitation, the measurements are corrected for undercatch as indicated in the Methods section. (b) 2m air temperature. (c) Relative humidity. (d) Wind speed. (e) Incoming shortwave radiation. The agreement between the downscaled reanalysis and the observations is quantified using mean error (ME), root-mean squared (RMSE) and coefficient of determination (r^2).

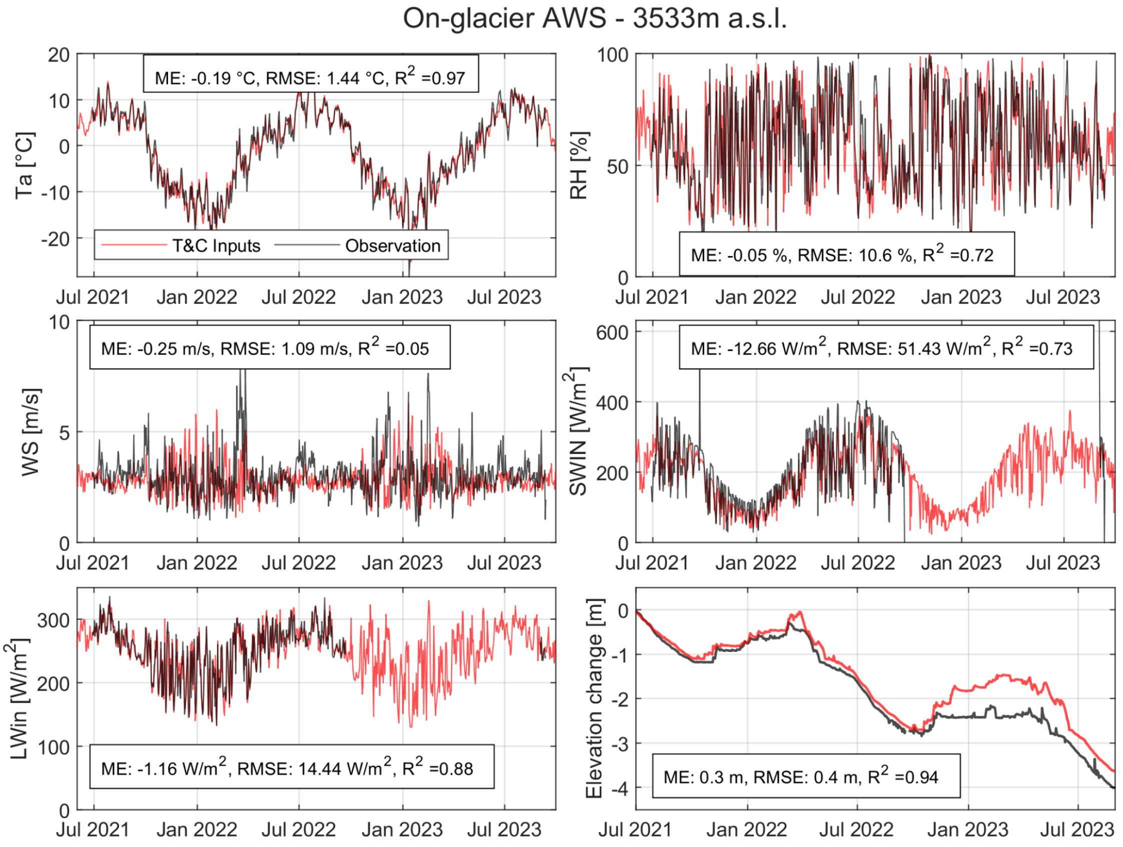

Figure S5. Evaluation of ERA5-Land downscaled and bias-corrected meteorological forcing at the location of the on-glacier AWS which has been operating since June 2021. All variables are shown at daily time scale. (a) 2m air temperature. (b) Relative humidity. (c) Wind speed. (d) Incoming shortwave radiation. (e) Incoming longwave radiation. (f) Surface elevation change measured by an ultrasonic depth gauge. The agreement between the downscaled reanalysis and the observations is quantified using mean error (ME), root-mean-squared error (RMSE) and coefficient of determination (r^2).

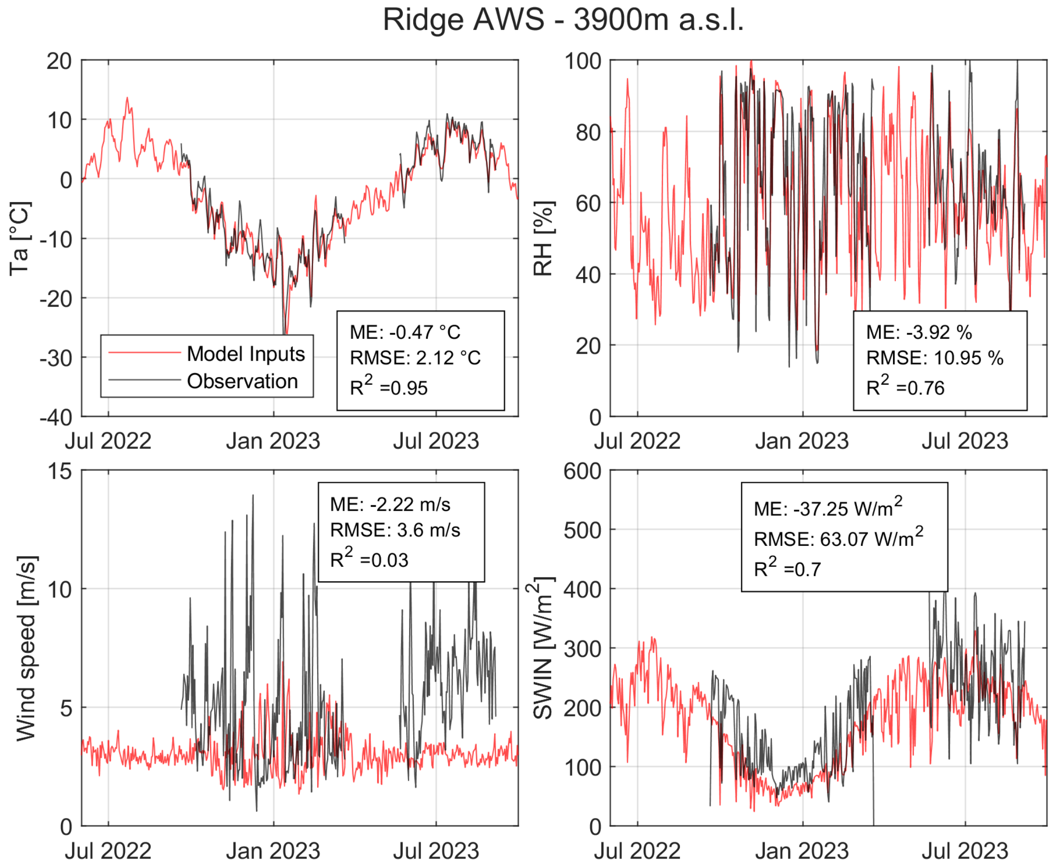

Figure S6. Evaluation of ERA5-Land downscaled and bias-corrected meteorological forcing at the location of the ridge AWS which has been operating since September 2022. All variables are shown at daily time scale. (a) 2m air temperature. (b) Relative humidity. (c) Wind speed. (d) Incoming shortwave radiation. The agreement between the downscaled reanalysis and the observations is quantified using mean error (ME), root-mean squared (RMSE) and coefficient of determination (r^2).

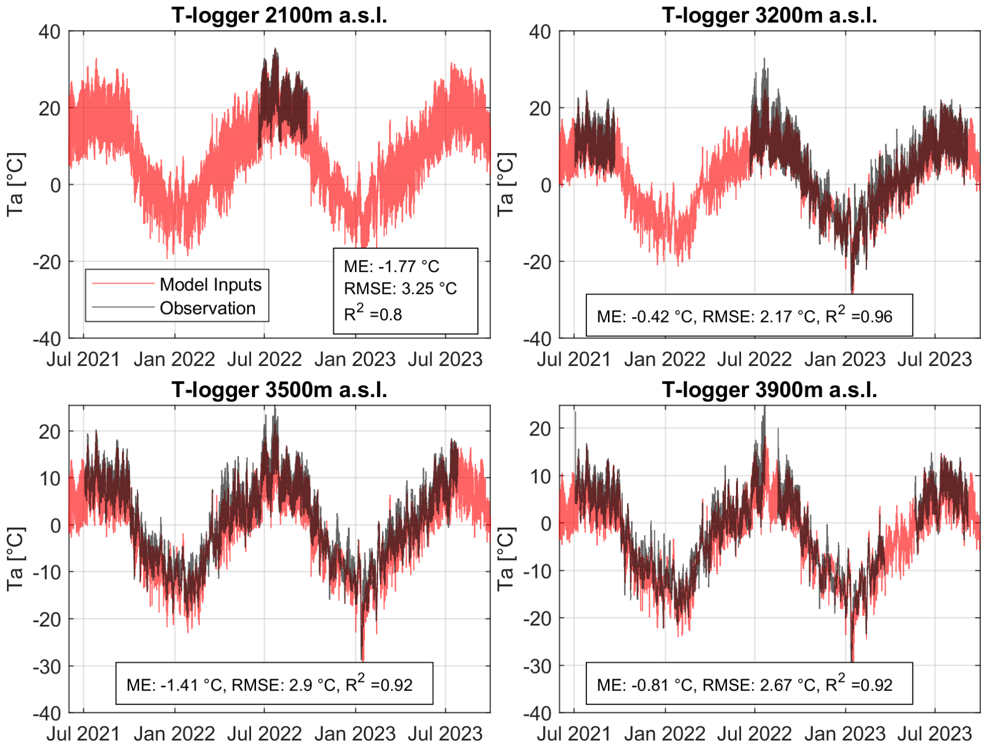

Figure S7. Evaluation of ERA5-Land downscaled and bias-corrected meteorological forcing at the location of 2m air temperature sensors equipped with radiation shields. All variables are shown at an hourly time scale. The agreement between the downscaled reanalysis and the observations is quantified using mean error (ME), root-mean squared (RMSE) and coefficient of determination (r^2).

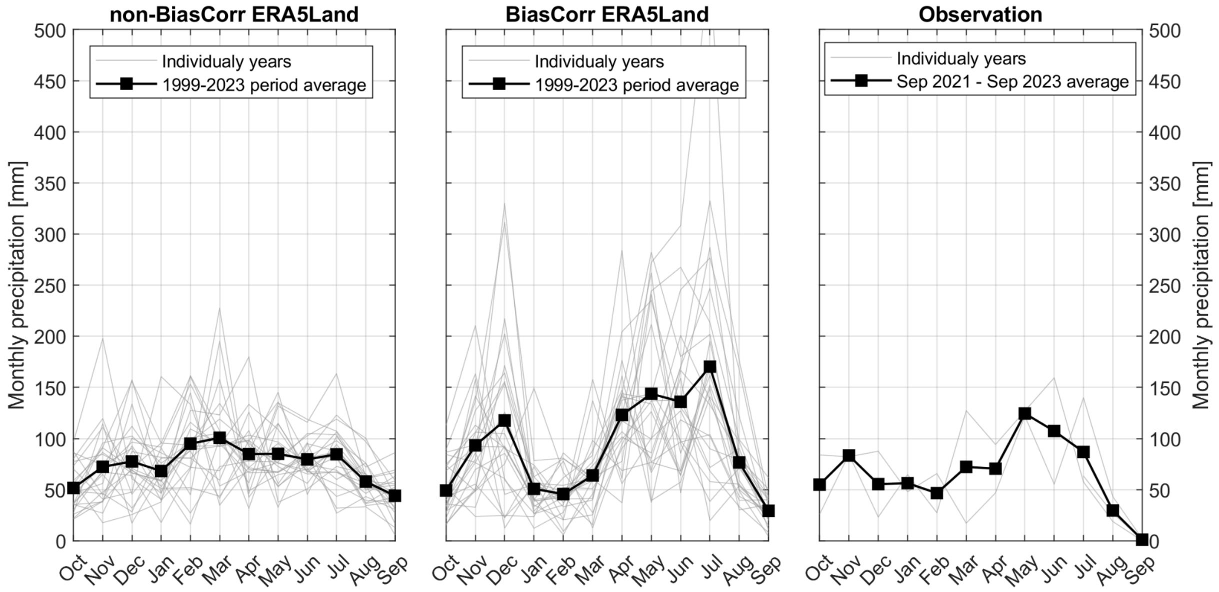

Figure S8. Precipitation estimated by ERA5-Land at the location of the pluviometer station (3369 m a.s.l.) from 2000 to 2023 for each month of the year before (a) and after (b) applying a bias-correct correction based on the undercatch corrected precipitation measurement.

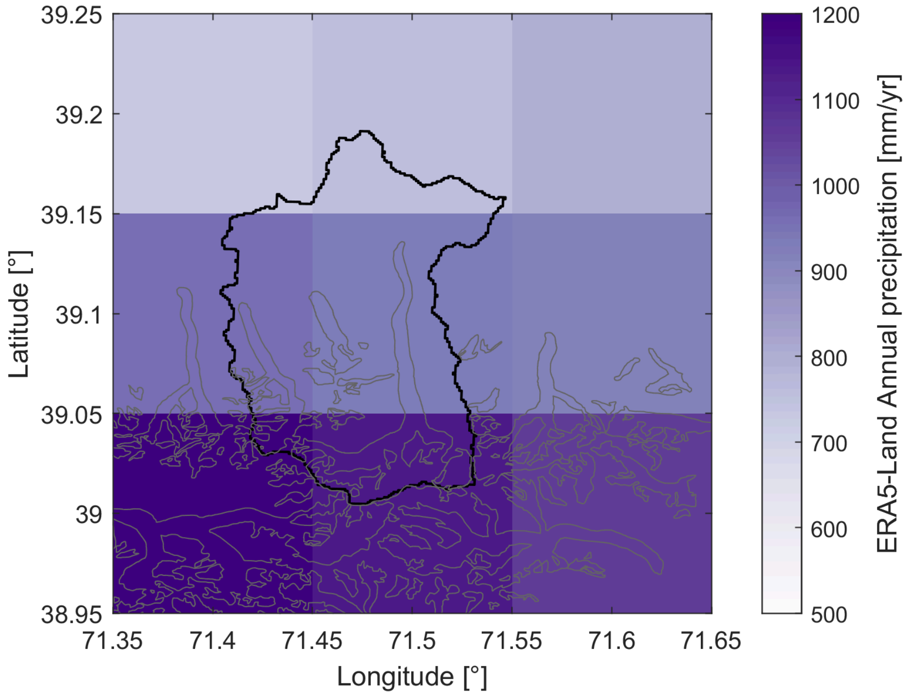

Figure S9. Spatial variability of ERA5-Land precipitation around our catchment of interest, delineated with a solid black line. Glacier outlines from the RGI 6.0 inventory are shown as solid grey lines. The annual precipitation amounts shown here are estimated by the non-corrected ERA5-Land product and are averaged over the 2000-2023 period.

3. Supplementary methods 3: remote sensing of glacier and snow changes

3.1 High-resolution snow cover mapping

We used all available Landsat-5/7/8/9 and Sentinel-2 scenes from October 1999 to September 2023 to derive snow cover maps at high spatial resolution (30m). The scenes were downloaded using the Microsoft Planetary Computer, setting a maximum cloud cover filter of 10% and further manually discarding all scenes with clouds present within our catchment outlines. Mapping snow over glacierized areas is more challenging than over other land cover types, such that we used two different classifying methods over and outside of clean-ice areas. We delineate clean-ice areas as areas for which the NDSI value averaged over the 2000-2023 period exceeds 0.7 (Fig. S10), thus limiting edge effects that could arise from using existing glaciers and debris outlines. An example of snow cover mapping is provided in Fig. S11, chosen for a date where the snow does not cover all of the clean ice areas. We discarded areas affected by cast shadows with the Matlab function *castshadow.m* developed by Wolfgang Schwanghart, which computes cast shadows from the digital elevation model, azimuth and elevation angles of the satellite. An example of snow cover mapping of a scene affected by cast shadows is shown in Fig. S12.

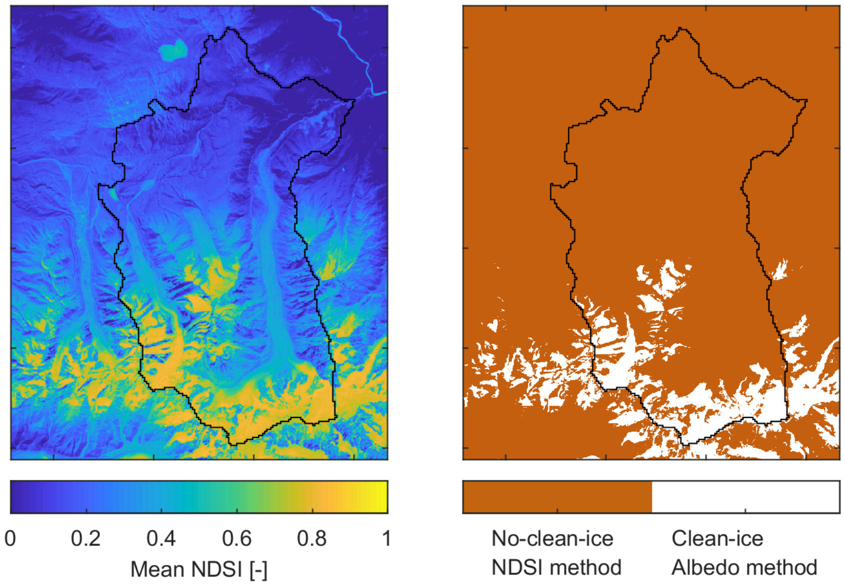

Figure S10. Mean NDSI computed from all available Sentinel-2 and Landsat 5/7/8/9 for the period 1999-2023 (left). Result of the clean-ice area classification after applying a mean NDSI threshold of 0.7, indicating which method will be used to distinguish snow from other land covers (right).

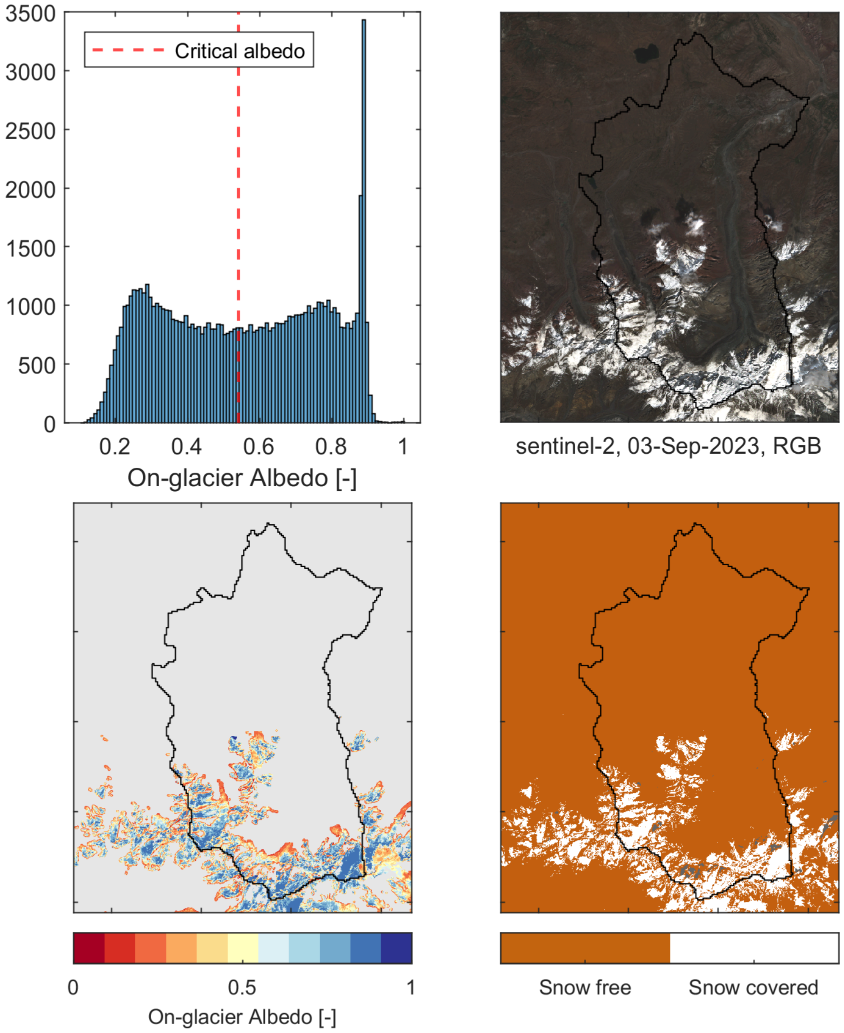

Figure S11. Processing of high-resolution snow cover maps. Determination of critical albedo threshold over clean-ice areas using the Otsu algorithm (top-left). Sentinel-2 scene acquired on 3 September 2023 and used in this example, shown in RGB (top-right). Surface albedo derived over clean-ice areas (bottom-left). Final snow cover map (bottom-right). The catchment outlines used in this study are shown as a solid black line. Areas for which no snow classification could be done are displayed in dark grey.

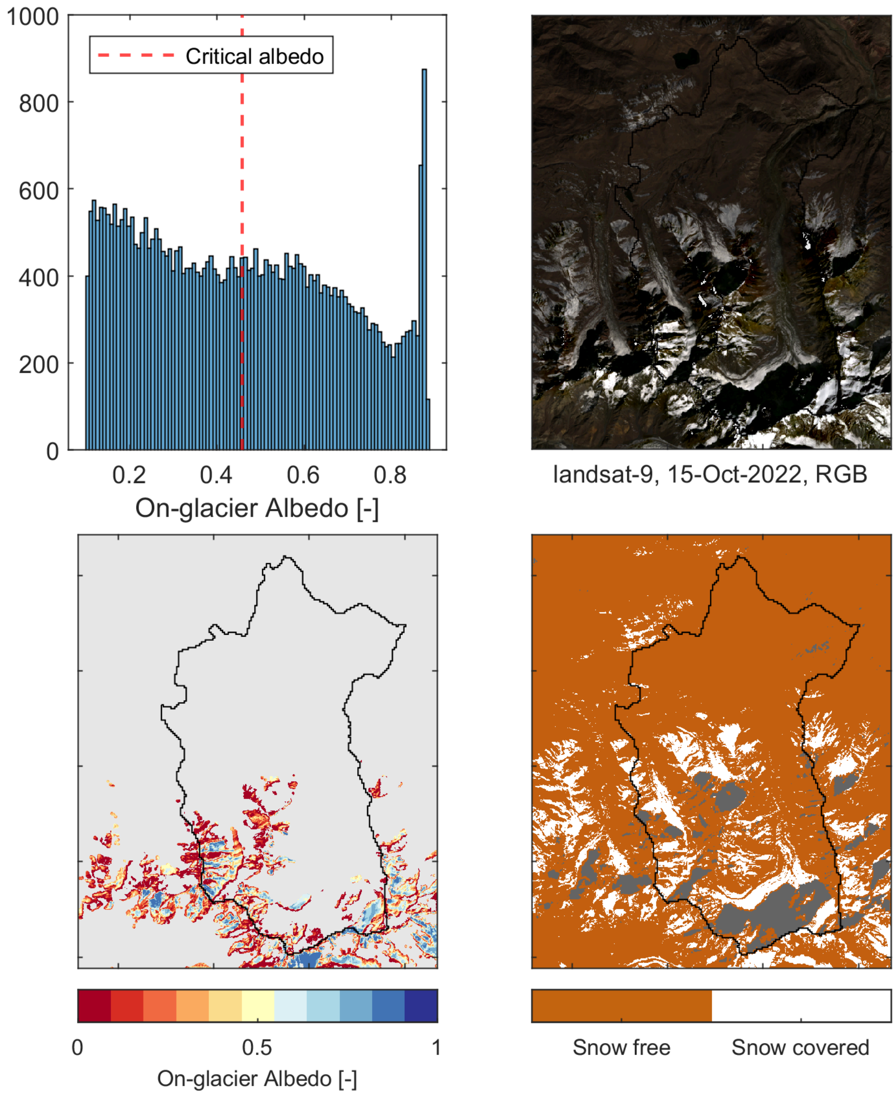

Figure S12. Processing of high-resolution snow cover maps. Determination of critical albedo threshold over clean-ice areas using the Otsu algorithm (top-left). Landsat-9 scene acquired on 15 October 2022 and used in this example, shown in RGB (top-right). Surface albedo derived over clean-ice areas (bottom-left). Final snow cover map (bottom-right). The catchment outlines used in this study are shown as a solid black line. Areas for which no snow classification could be done are displayed in dark grey.

3.2 Spatially resolved snow depth from very high-resolution stereo imagery

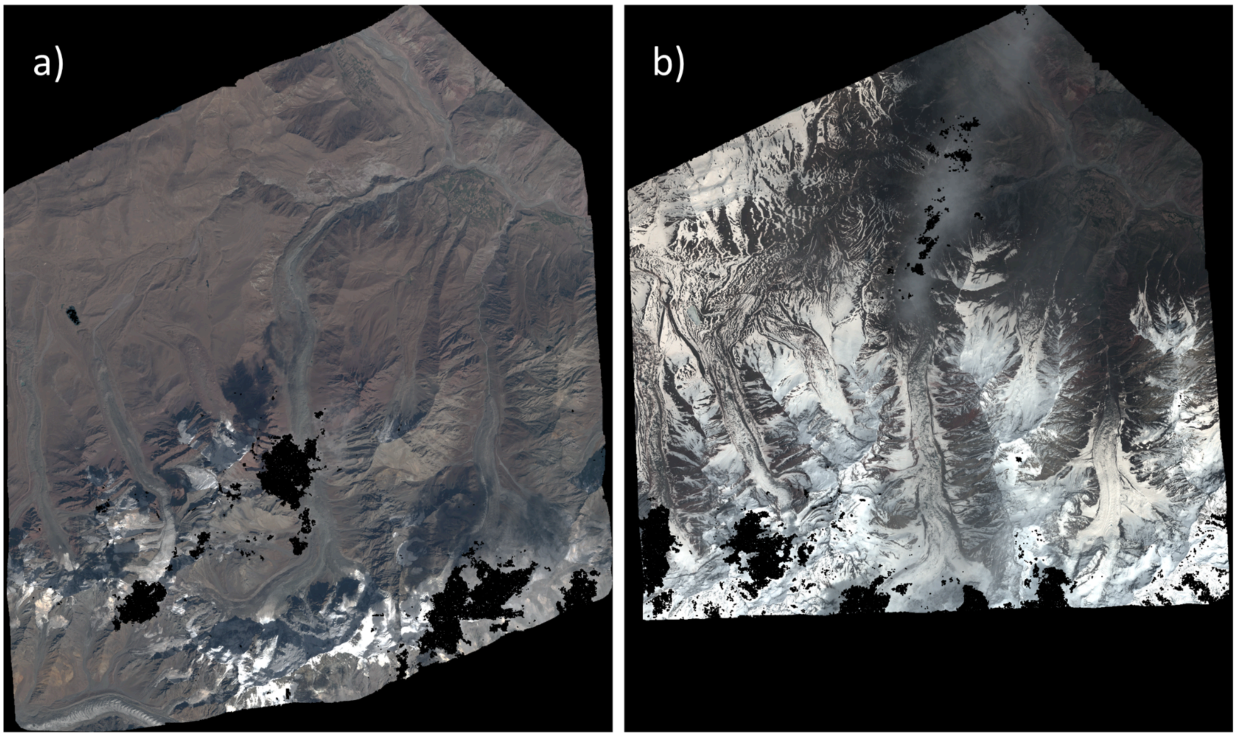

Figure S13. Pléiades stereo images acquired over the Kyzylsu study area. The first scene (a) was acquired on September 24th 2022 during snow-free conditions, and the second one (b) was acquired on May 23rd 2023 when a large proportion of the catchment was snow-covered. Areas in black indicate areas not covered by the tasking or affected by clouds.

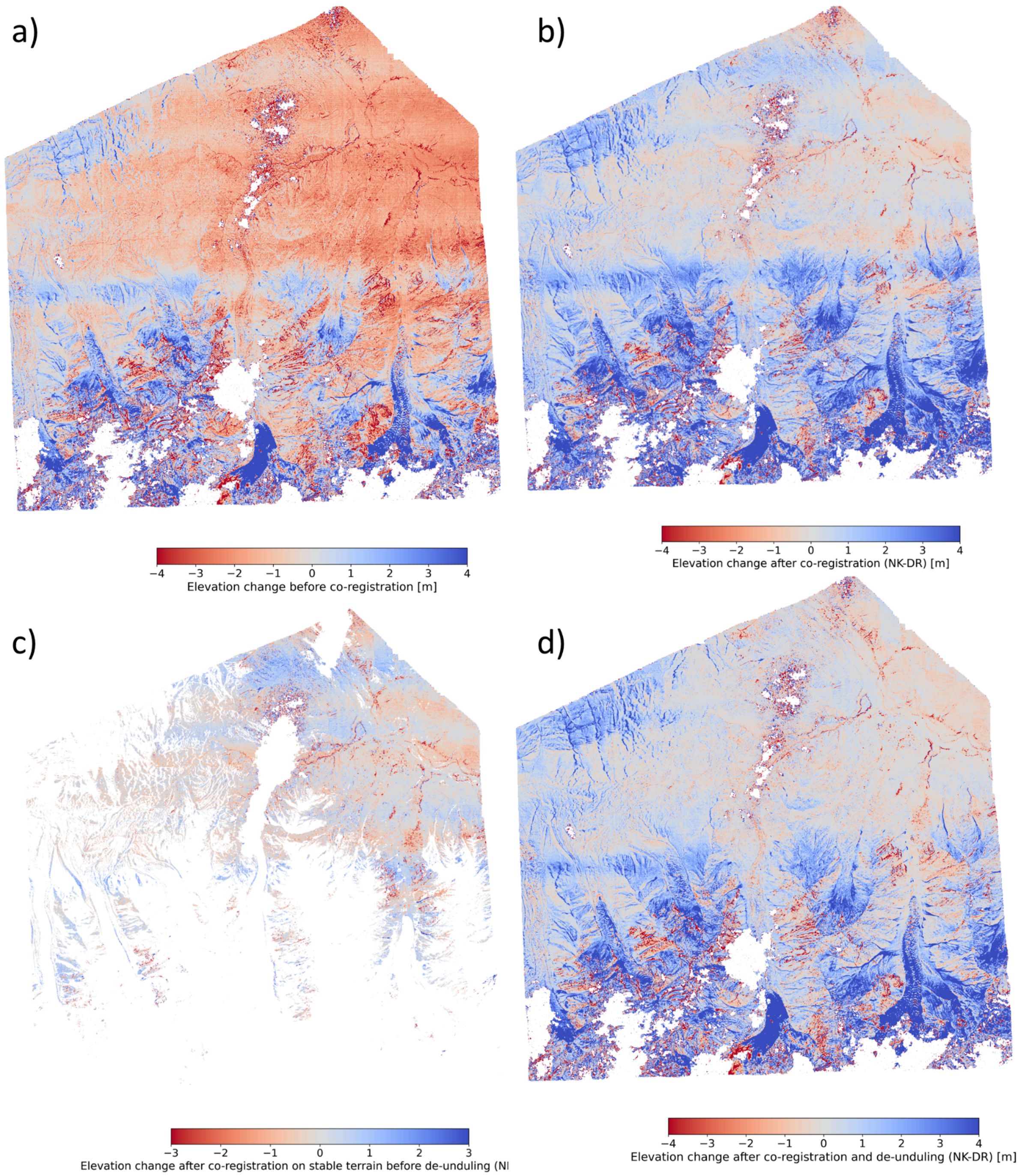

Figure S14. Pleiades DEMs co-registration and differencing. a) Elevation change derived from DEMs differencing before their co-registration. b) Elevation change derived after applying a Nuth & Kaab co-registration with a deramping (tilt correction). c) Elevation change on stable terrain as in b). (d) Elevation change after applying a jitter correction on the elevation change shown in b), using the stable terrain mask shown in c).

4. Supplementary methods 4: Model set-up

4.1 Land covers

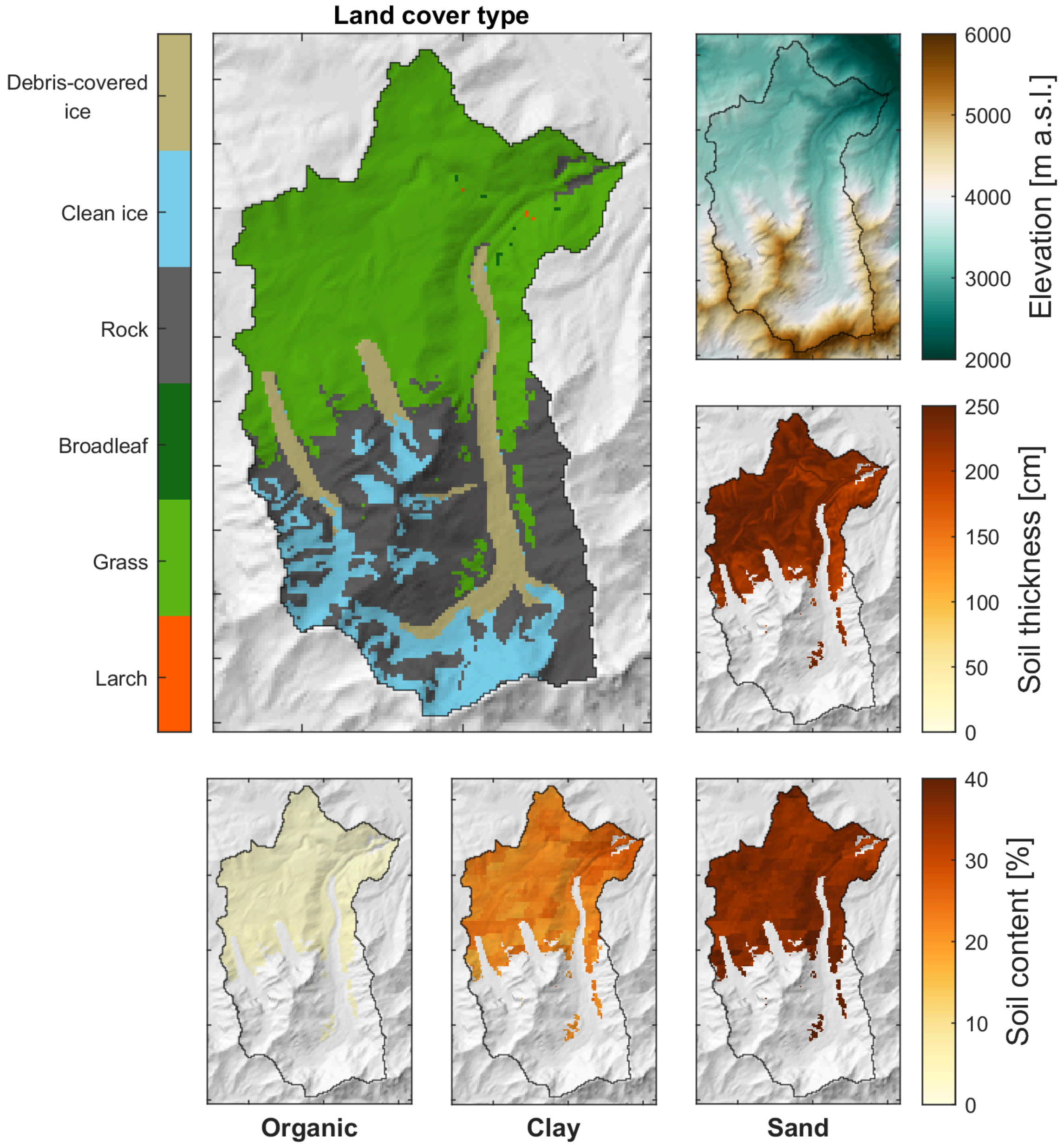

Figure S15. Distributed model set up of land cover areas. The catchment outlines are shown as a black solid line. All spatial variables shown here are at 100m spatial resolution. (a) Land cover types from PROBA-V. (b) Surface elevation from AW3D DEM. (c) Soil thickness derived from the SOILGRID product. (d) Organic matter content with the soil column. (e) Clay content within the soil column. (f) Sand content within the soil column.

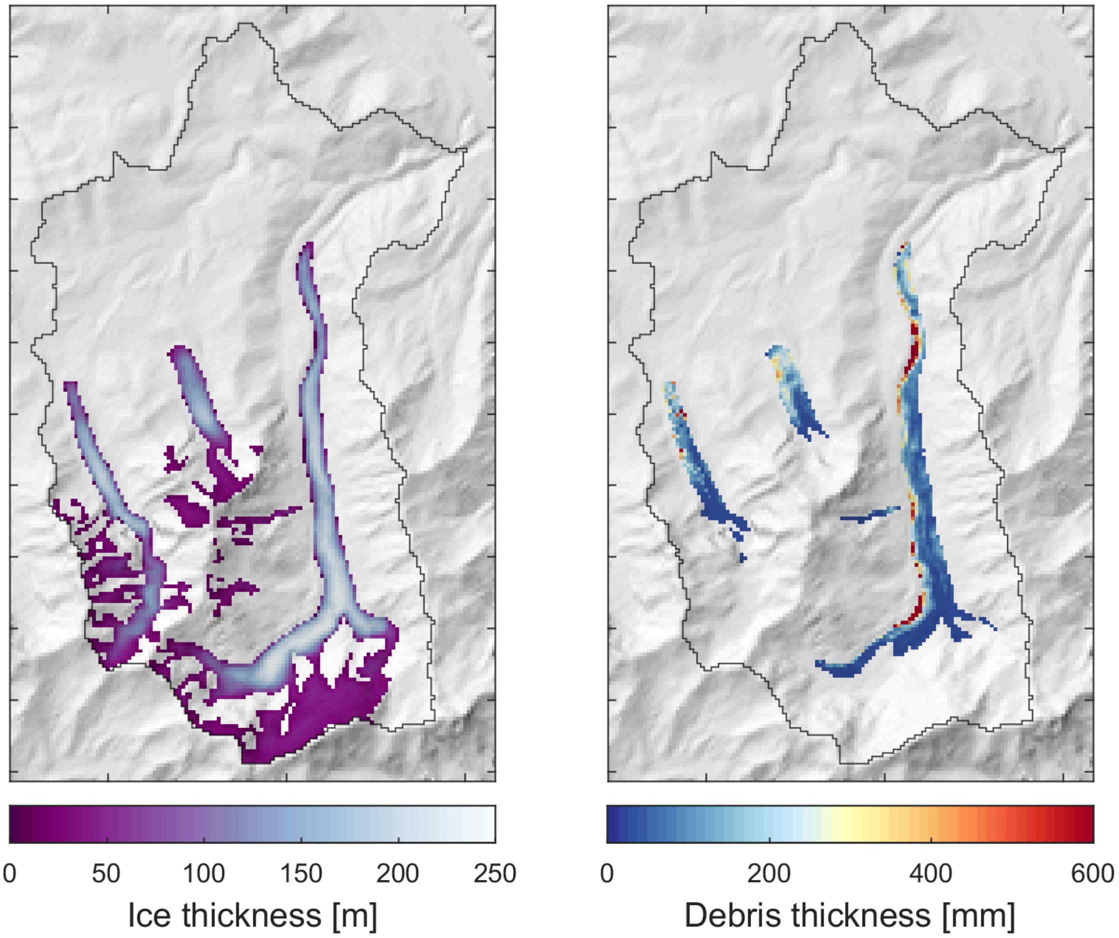

Figure S16. Distributed model set up of glacier and debris-covered areas. The catchment outlines are shown as a black solid line. (a) The ice thickness is from Farinotti et al. 2019 [1], but is only indicative here as the ice thickness used for the simulation was artificially increased by 400 meters to prevent the disappearance of ice on some pixels that could occur when not accounting for glacier flow. (b) Distributed debris thickness used for model simulations, which we assumed to be constant in time.

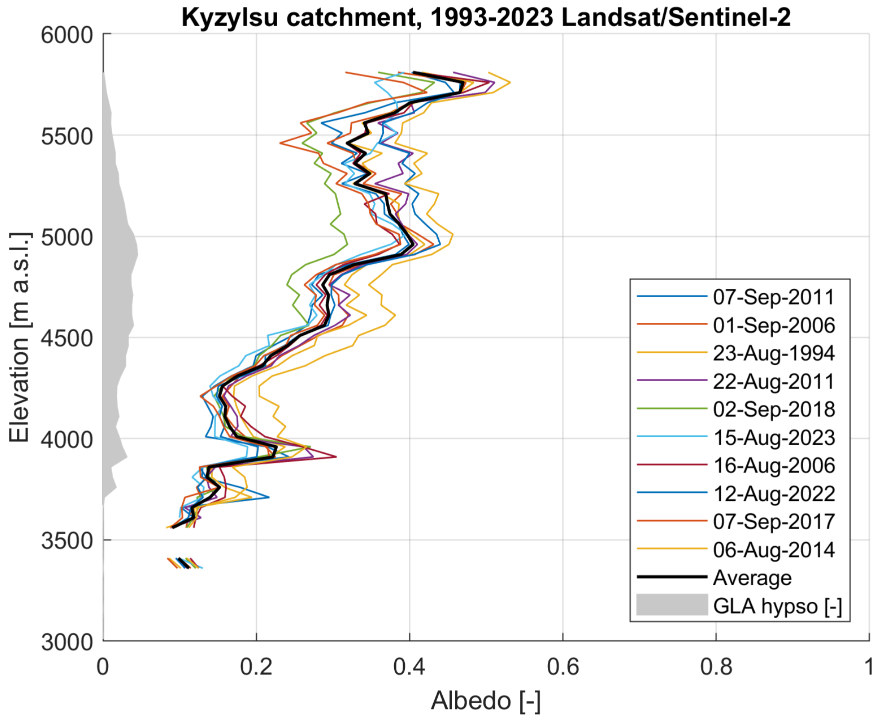

Figure S17. Glacier surface albedo observed per 100-m elevation band for the 10 least snow-covered available Landsat and Sentinel-2 scenes, whose corresponding dates are indicated in the legend. The average glacier albedo per elevation band is shown as a thick black line and is used to set the distributed bare-ice albedo parameter values. The glacier hypsometry of the catchment is shown as a grey-shaded area on the left of the figure. Debris-covered areas were excluded from this analysis.

4.2 Initial conditions

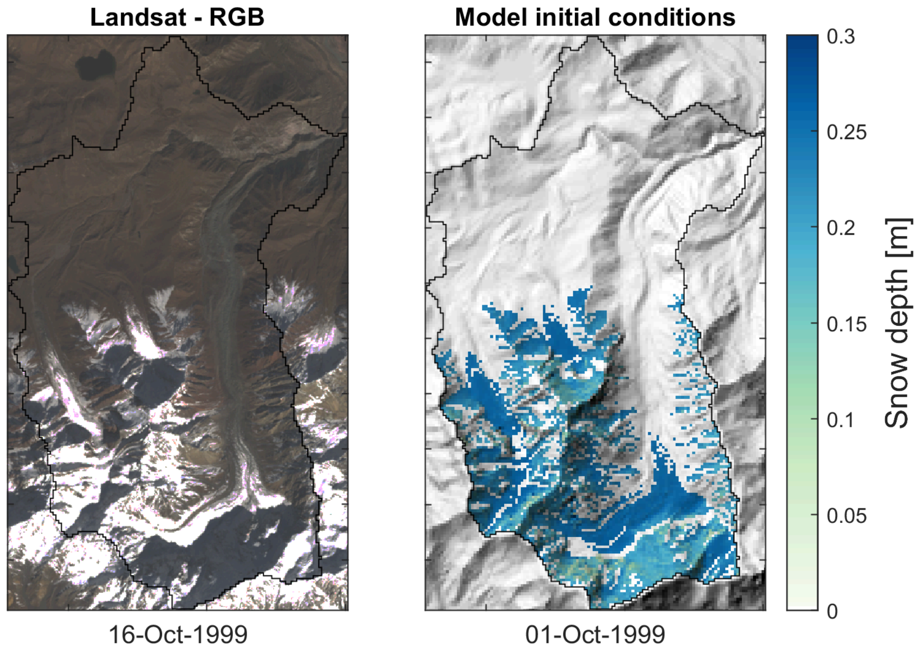

Figure S18. Initial snow cover and snow depth based on the Landsat-7 scene acquired on October 16th 1999. Snow cover and snow surface albedo in shaded areas were set to the

non-shaded area average of the corresponding elevation band. In the absence of snow depth information, we set the initial snow depth to 30 centimeters or to the maximum snow holding depth according to the avalanche routine if the latter is below 30 centimeters.

4.3 Glacier dynamics

Glacier flow was not accounted for in this study, as glacier areas and glacier surface elevation did not change substantially through that period. We compared glacier outlines from the RGI 6.0 inventory which are based, at this location, on satellite images from 16 September 2000, with glacier outlines re-delineated by Kneib et al. 2023 [2] based on a Pleiades ortho-image acquired on 24 September 2022 (Fig. S19). Apart from Muzgagzy Glacier (the longest glacier located on the eastern side of our catchment), there have been minimal changes in the extent of glacier ablation areas. Furthermore, the glacier surface elevation has on average not changed substantially, despite thinning and thickening both happening on several glaciers (Fig. S20). The surge behavior of these glaciers is not uncommon in this region [3] but would complicate efforts aiming at representing ice dynamics in the modeling framework.

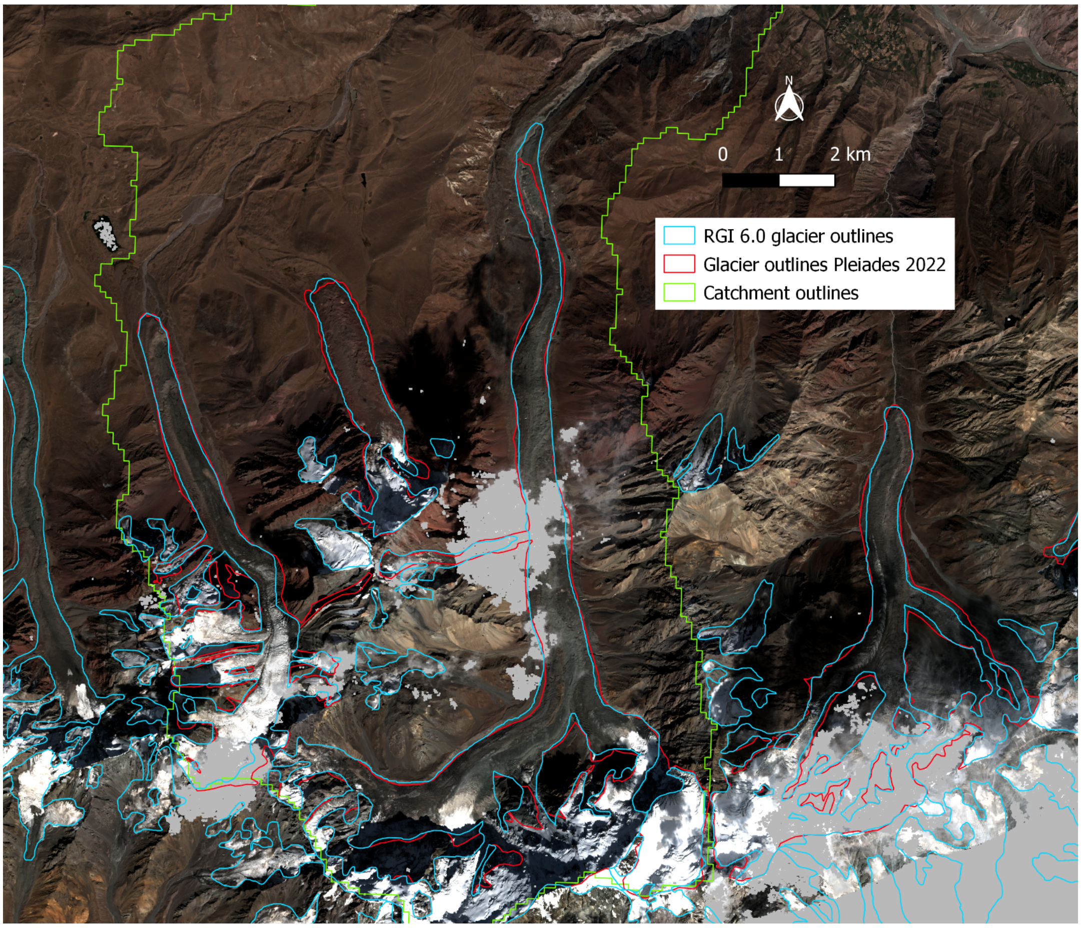

Figure S19. Comparison between glacier outlines from the RGI 6.0 corresponding to the date of 16 September 2000 and glacier outlines manually delineated from a Pleiades image acquired on 24 September 2022 [2]. Only the main glaciers were delineated from the 2022 Pleiades image. The catchment outlines used in this study are shown as a solid green line. Grey areas correspond to data gaps in the Pleiades ortho-image.

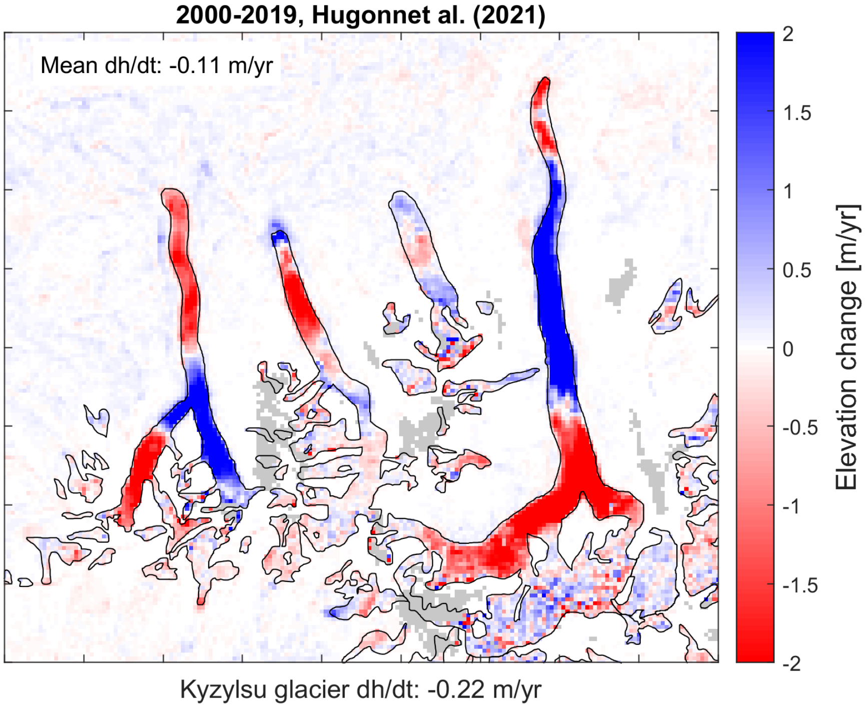

Figure S20. Elevation change derived from ASTER times-series of digital elevation model (Hugonnet et al. 2021 [3]) over our study area, for the period 2000-2019. The mean elevation changes indicated in the figure were computed within the glacier outlines of the RGI 6.0 inventory.

4.4 Gravitational redistribution

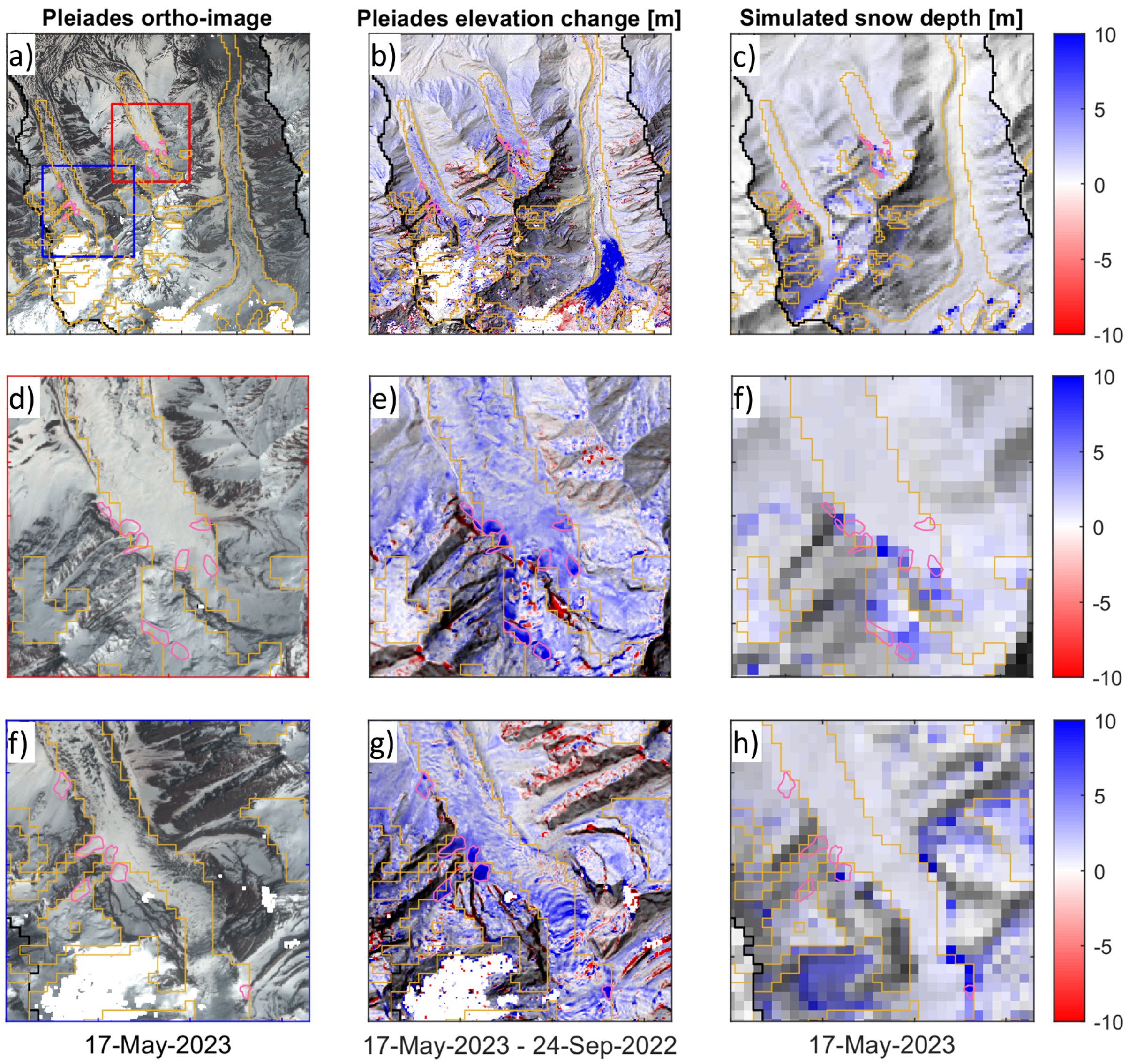

Figure S21. Evaluation of avalanche deposits, comparing surface elevation change derived from the subtraction of two Pleiades DEMs corresponding to the date of 24 September 2022 (snow-free) and 17 May 2023 (snow-covered) with distributed snow depth simulations. Pink outlines indicate avalanche deposits manually delineated from the ortho-image and surface elevation change. (a) Pleiades ortho-image acquired on 17 May 2023. The red and blue rectangles indicate the zoomed areas displayed in panels d-f and g-i respectively. (b) Surface elevation change derived from Pleiades differencing after DEMs co-registration. White areas correspond to data gaps. (c) Snow depth simulated by our model at the date corresponding to the snow-covered Pleiades acquisition, 17 May 2023. The avalanche parameters used are a = 0.12 and C = 145.

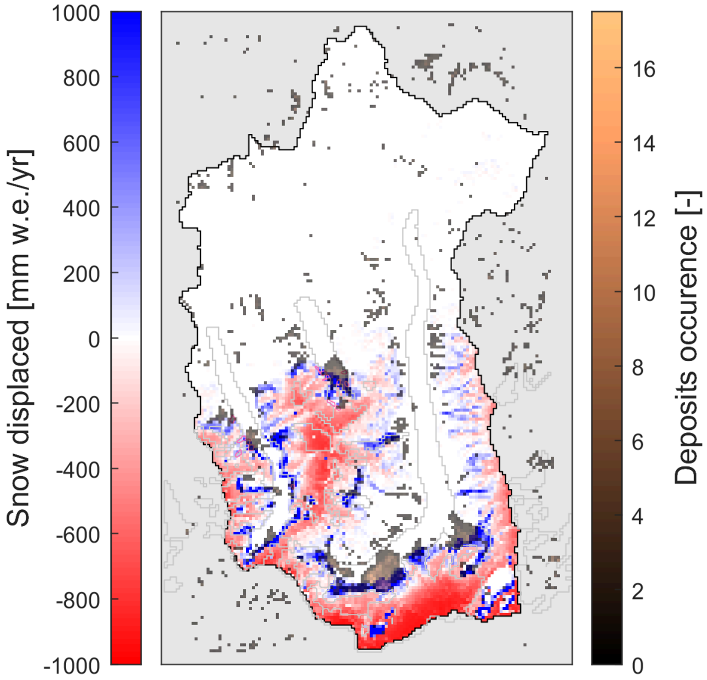

Figure S22. Comparison of the snow displaced by avalanches in the model with avalanche deposits observed in Sentinel-1 images, from 2017 to 2023. Glacier outlines are indicated in grey. Negative values indicate that snow has been removed from the corresponding pixels (shown in red), while positive values (blue areas) indicate a supply of snow from avalanches. The deposit outlines delineated from Sentinel-1 should therefore only be compared with blue areas.

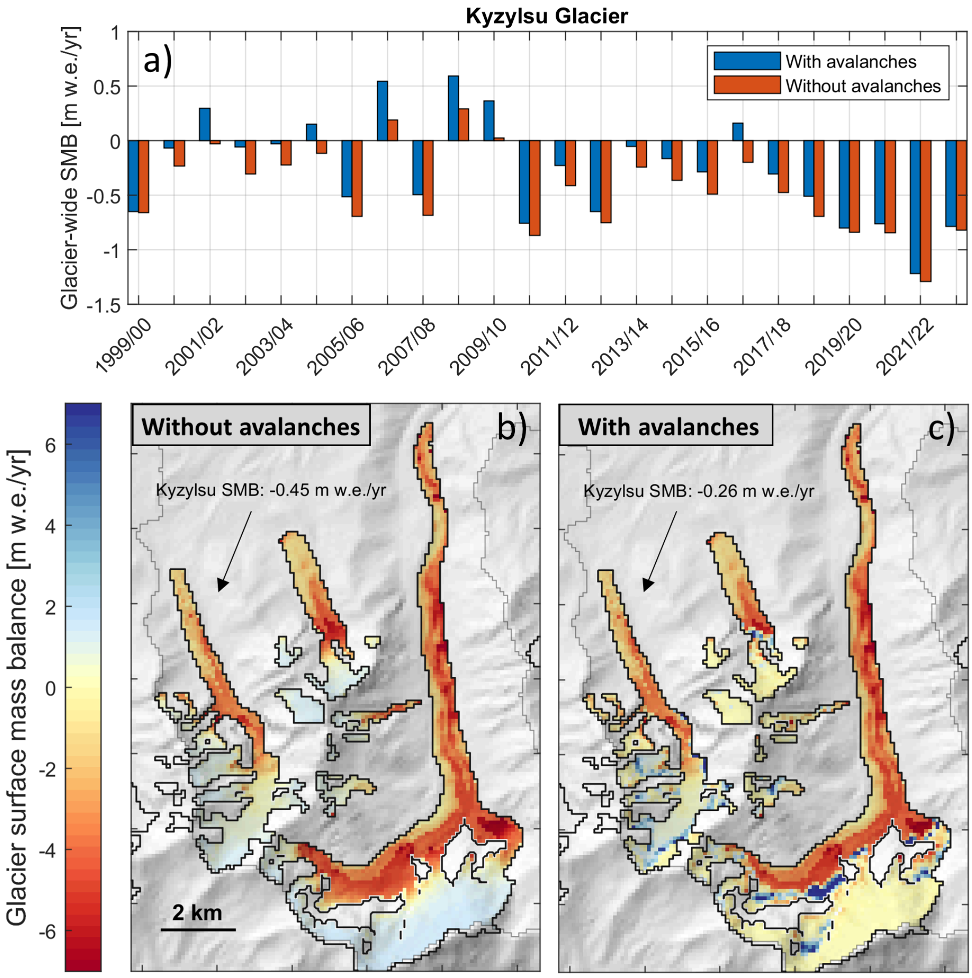

Figure S23. The sensitivity of glacier mass balance to the representation of avalanches. (a) Annual time-series of glacier-wide surface mass balance for Kyzylsu Glacier, comparing simulations without (red) and with (blue) including snow gravitational redistribution. Spatially distributed surface mass balance of Kyzylsu Glacier for the period 2000-2023 without (b) and with (c) including snow gravitational redistribution. Including the representation of snow redistribution increases Kyzylsu's glacier mass balance and its inter-annual variability, from -0.45 ± 0.38 to -0.26 ± 0.47 m w.e./y.

4.5 Model limitation: lack of firn representation

Our model uses a 2-layer snowpack model, featuring a 6 mm thick surface skin layer that enables energy exchange with the atmosphere and heat transfer within the snowpack, but it does not include a firn layer. Using a more detailed snowpack model with a representation of firn would have been hampered by the absence of ground observation in the accumulation area to validate the model results. At Kyzylsu Glacier, as in most high elevation sites in the Pamirs, the accumulation area of the glacier is not accessible on foot (icefall and avalanche danger) and the usage of helicopters is very challenging in Tajikistan. Long-term firn modeling was conducted by Kronenberg et al. (2022) [5] at Abramov Glacier, which is located only 60 kilometers north of Kyzylsu Glacier, for the period 1968-2020. They found that periods of negative mass balance led to a loss of firn pore space and a decrease in

internal accumulation (refreezing and storage of water in the firn). They report decadal internal accumulation ranging from 0.08 m w.e. a^{-1} in 2011-2020 to 0.13 m w.e. a^{-1} in 1988-1998. The decadal differences in internal accumulation (+/- 0.05 m w.e. a^{-1}) are relatively small compared to our simulated changes in mass inputs at Kyzylsu Glacier due to snowfall (-0.31 m w.e./yr) and avalanche changes (-0.13 m w.e./yr). While changes in firn regimes might have occurred at Kyzylsu Glacier in the last two decades, this is likely to be a minor process compared to precipitation changes.

5. Supplementary methods 5: model evaluation

We evaluated the model performance at the point scale, when compared with hydrometeorological ground stations, and at the distributed scale, when compared with remotely sensed observations. Point-scale evaluation was performed with surface albedo (Fig. S24), glacier ablation stakes (Fig. S25), glacier surface elevation change (Fig. S26), snow depth (Fig. S27), proglacial stream water level (Fig. S28). Model evaluation at the catchment (or glacier) scale was conducted against remotely-sensed snow cover fraction (Fig. S29-30), snow line altitude (Fig. S31-32) and glacier-wide geodetic mass balance for the period 2000-2019 (Fig. 5). We provide in Table S2 a summary the model performance metrics.

Table S2: Summary of model performance evaluated against ground and remotely-sensed observations, as quantified by the mean error (ME), root-mean squared error (RMSE) and coefficient of determination (R^2).

**Variable and unit**	**Scale**	**Station or satellite**	**ME**	**RMSE**	**R^2**	**Figure**
Surface albedo [-]	Point	AWS on-glacier (3579 m a.s.l.)	0	0.17	0.72	S24
Surface albedo [-]	Point	Pluviometer (3369 m a.s.l.)	-0.10	0.17	0.83	S24
Glacier ablation [m w.e.]	Point	Ablation stakes	+0.23	0.48		S25
Surface elevation [m]	Point	AWS on-glacier (3579 m a.s.l.)	+0.30	0.40	0.94	S26
Snow depth [m]	Point	Pluviometer (3369 m a.s.l.)	+0.03	0.11	0.92	S27
Snow depth [m]	Point	Alpine village (3201 m a.s.l.)	-0.03	0.10	0.92	S27
Snow depth [m]	Point	On-glacier (3695 m a.s.l)	+0.14	0.21	0.89	S27
Stream level [cm]	Point	Alpine village (3200 m a.s.l.)			0.90	S28
Snow cover fraction [-]	Dist.	MODIS	-0.039	0.13	0.89	S29-30
Snow cover fraction [-]	Dist.	Landsat 5-7-8-9 and Sentinel-2	+0.049	0.11	0.94	S29-30
Snow line altitude [m]	Dist.	MODIS	60	370	0.77	S31-32
Snow line altitude [m]	Dist.	Landsat 5-7-8-9 and Sentinel-2	-45	390	0.92	S31-32

5.1 Point-scale evaluation

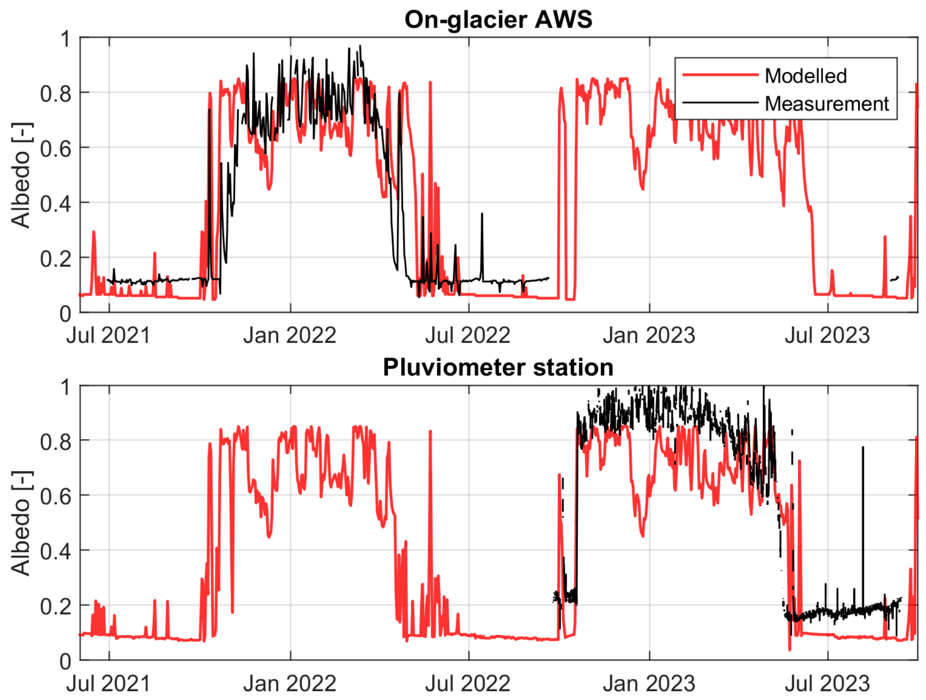

Figure S24. Evaluation of simulated surface albedo against measurement at the on-glacier AWS (top) and pluviometer station (bottom). The measured albedo was computed as the ratio of outgoing and incoming shortwave radiation. Only measured and simulated albedo at 12:00 local time is displayed here for readability.

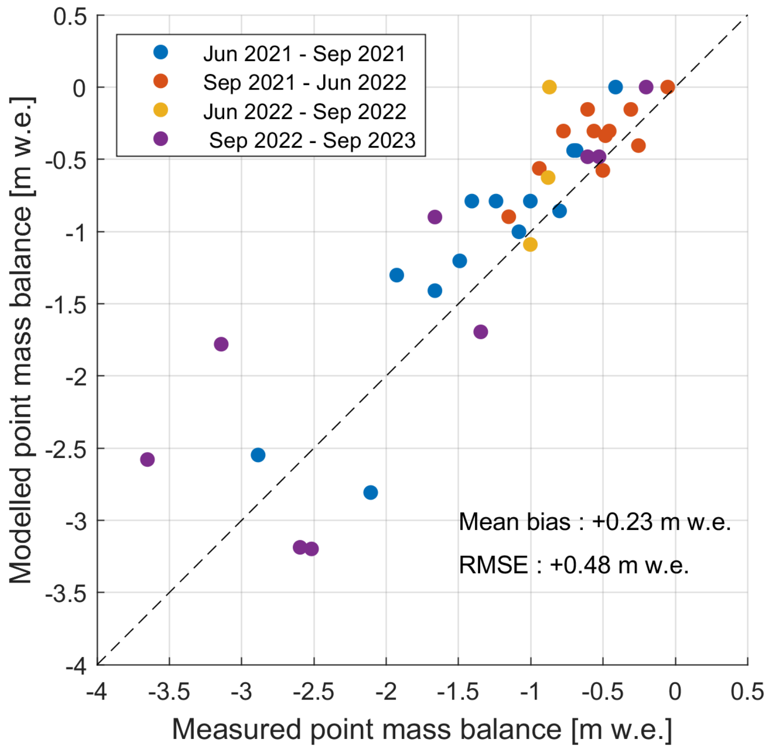

Figure S25. Evaluation of simulated glacier surface mass balance against measurement from ablation stakes set up at various places on the debris-covered portion of Kyzylsu Glacier (cf. Fig. 1). Each color represents a different mass balance period, related to our repeated visits at the study since 2021.

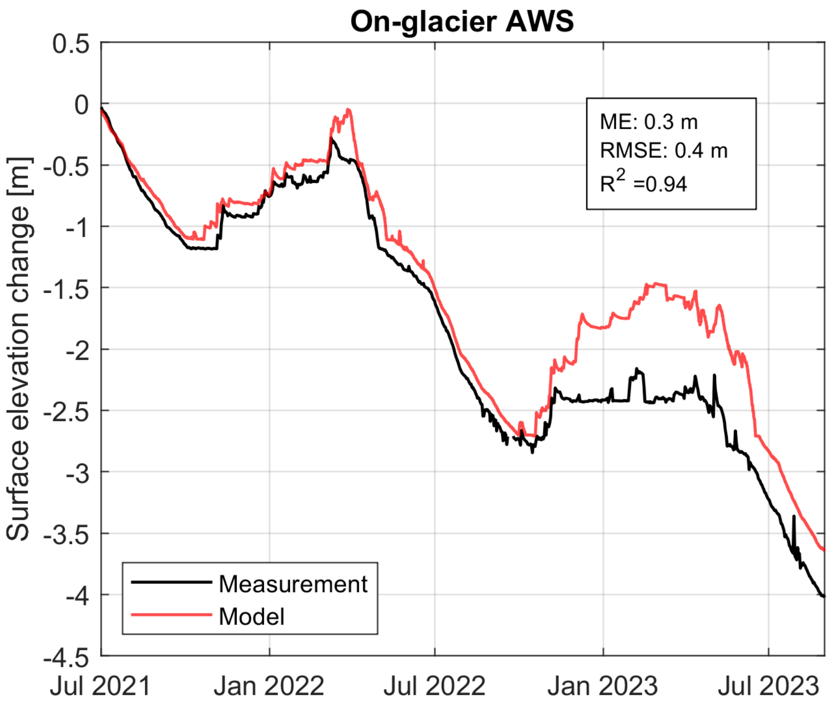

Figure S26. Evaluation of simulated glacier surface elevation change against measurement obtained from an ultrasonic depth gauge located near the on-glacier AWS on the debris-covered portion of Kyzylsu glacier. The agreement between the downscaled reanalysis and the observations is quantified using mean error (ME), root-mean-squared (RMSE) and coefficient of determination (r^2).

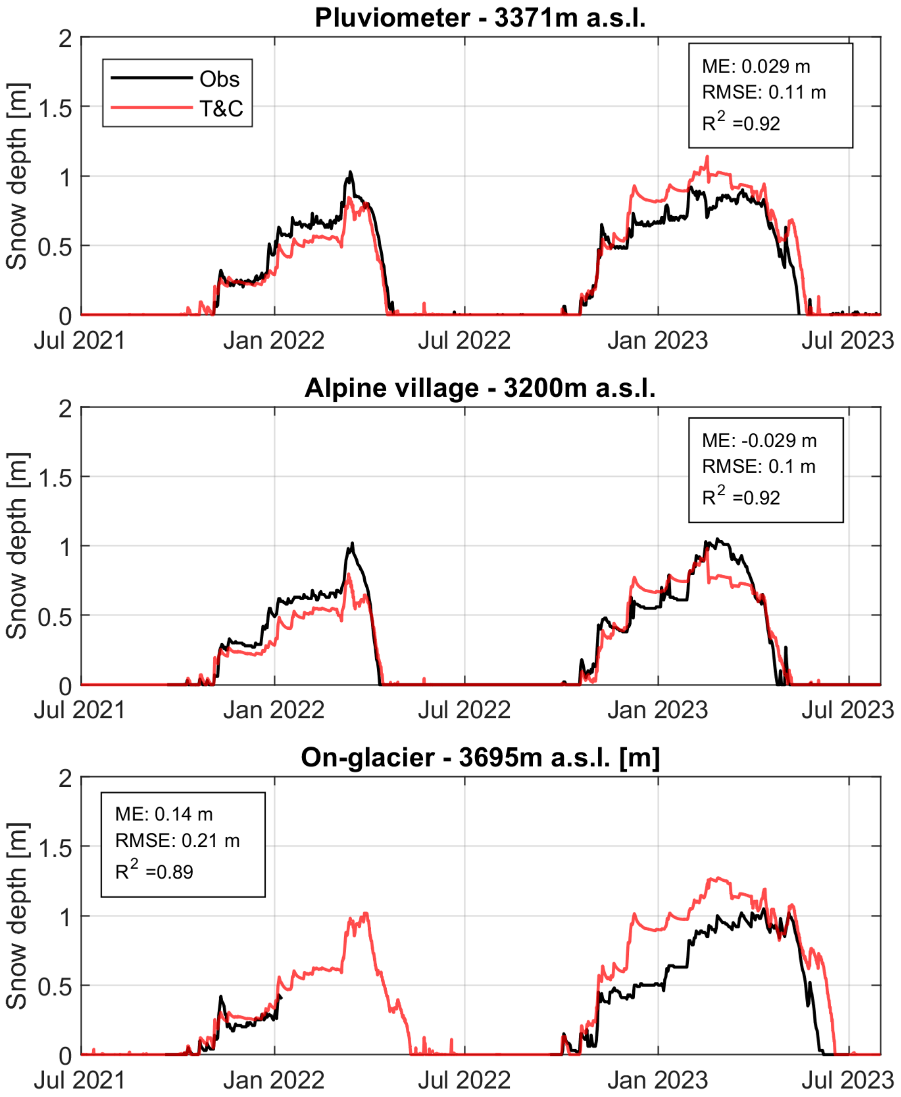

Figure S27. Evaluation of simulated snow depth measurement obtained from an ultrasonic depth gauge at the pluviometer station (top) or derived from daily photos of a graduated stake. The location of the time-lapse cameras is indicated in Table S1. The agreement between the downscaled reanalysis and the observations is quantified using mean error (ME), root-mean-squared (RMSE) and coefficient of determination (r^2). At the Alpine village location, the camera recorded daily pictures continuously from 24-Sep-2021 to 05-Sep-2023, including 15 days (2.4% of the period) for which snow depth could not be retrieved due to bad visibility or snow covering the camera lens. At the on-glacier location, the camera recorded continuously from 24-Sep-2021 to 09-Jan-2022 and from 19-Sep-2022 to 05-Sep-2023, including 6 days (1.3% of the period) for which snow depth could not be retrieved. The snow depth simulated at the pluviometer location (top) is underestimated in 2022 but overestimated in 2023 compared to observations. We hypothesise that the slight differences in modelled snowpack performance could be due to i) the meteorological forcing, even if ERA5-Land is bias-corrected, it might not fully represent the difference in meteorological conditions which occurred during these two winters ii) wind redistribution occurring during snowfall events, which is not represented in the model and could induce slight changes in the snow depth.

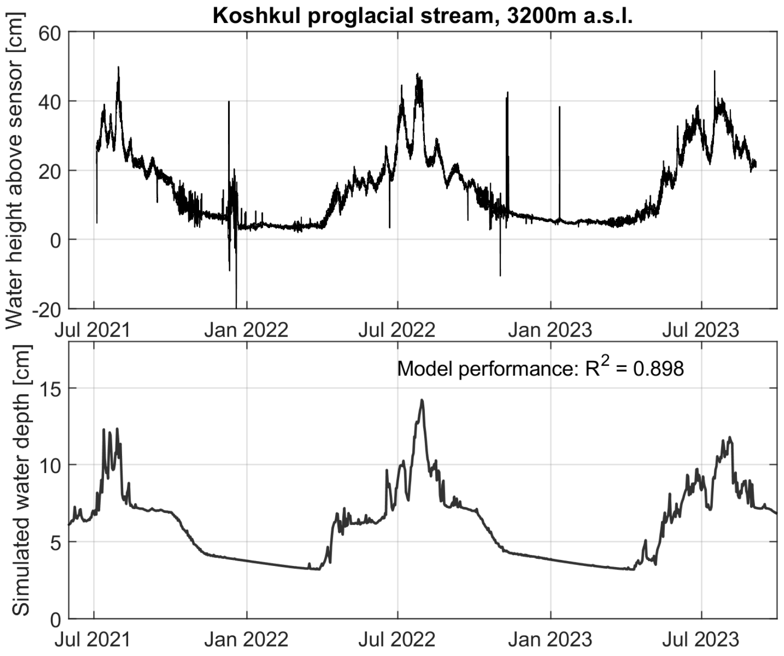

Figure S28: Model evaluation at the proglacial stream of Koshkul Glacier. (top) Measured water height above the HOBO level logger installed inside the stream. (bottom) Water depth in the channel simulated by the land-surface model at the pixel closest to the stream gauge.

5.2 Distributed scale evaluation

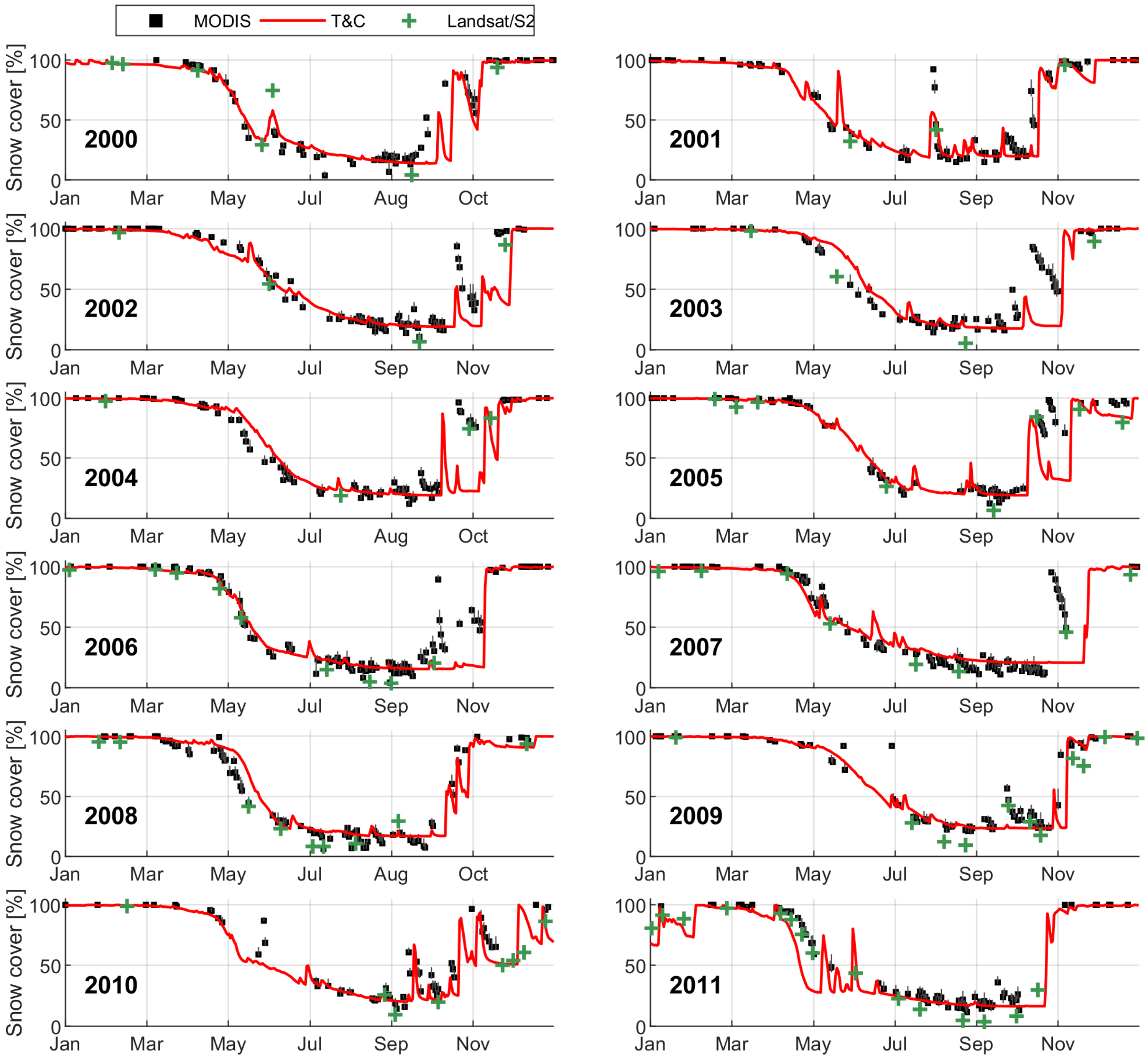

Figure S29. Evaluation of fractional snow cover against MODIS daily product, from 2000 to 2011 (2012-2023 shown in Fig. S30). It corresponds to the area of the Kyzylsu catchment covered by snow divided by the total catchment area. Only MODIS scenes with less than 10% of cloud cover are used in this comparison. Modeled fractional snow cover is shown on a daily scale. Vertical thin black bars above and below the black squares indicate the possible ranges of the MODIS snow cover obtained using different NDSI thresholds for the snow classification (0.25 - 0.40 - 0.45).

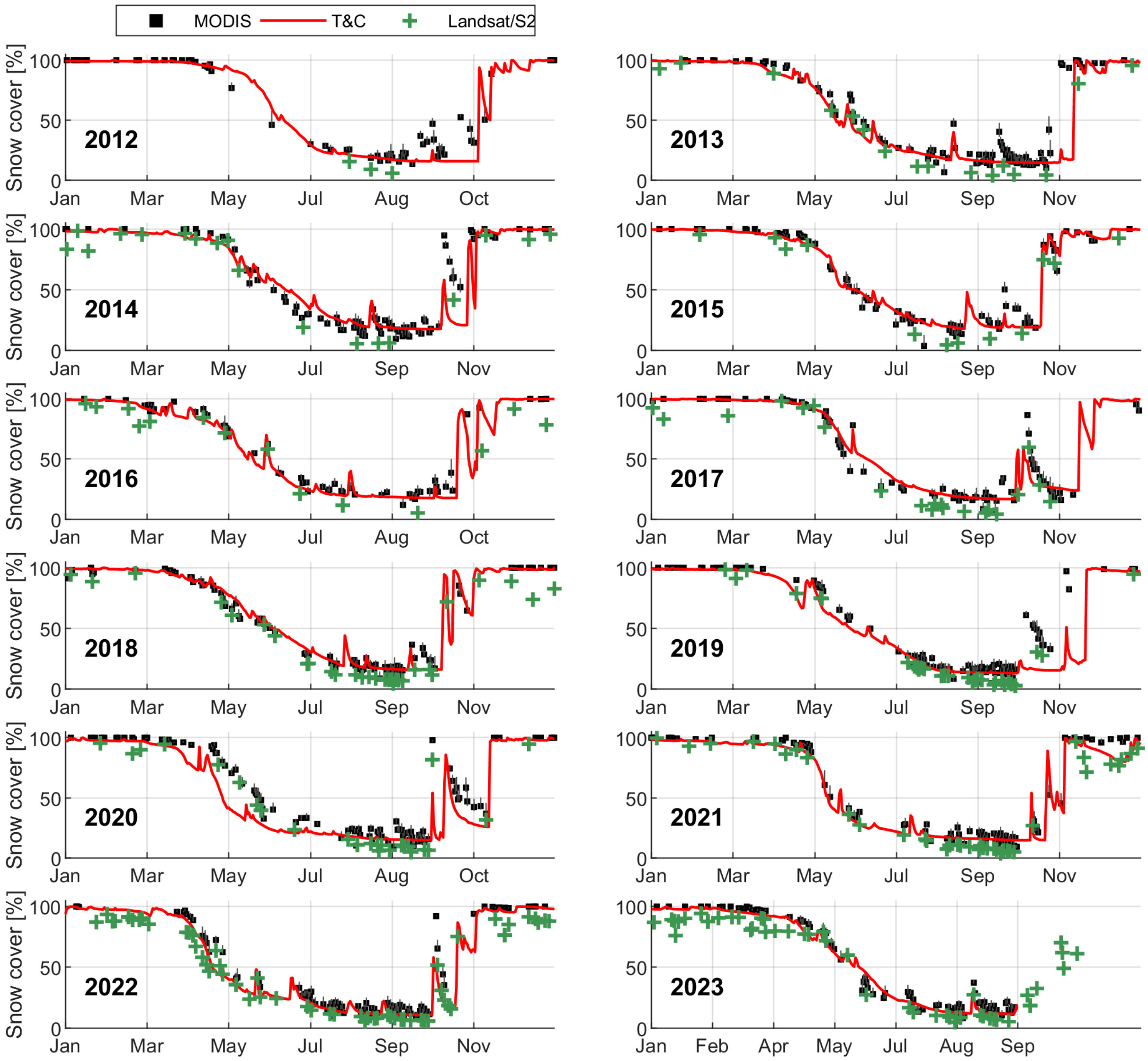

Figure S30. Evaluation of fractional snow cover against MODIS daily product, from 2012 to 2023. It corresponds to the area of the Kyzylsu catchment covered by snow divided by the total catchment area. Only MODIS scenes with less than 10% of cloud cover are used in this comparison. Modelled fractional snow cover is shown on a daily scale. Vertical thin black bars above and below the black squares indicate the possible ranges of the MODIS snow cover obtained using different NDSI thresholds for the snow classification (0.25 - 0.40 - 0.45).

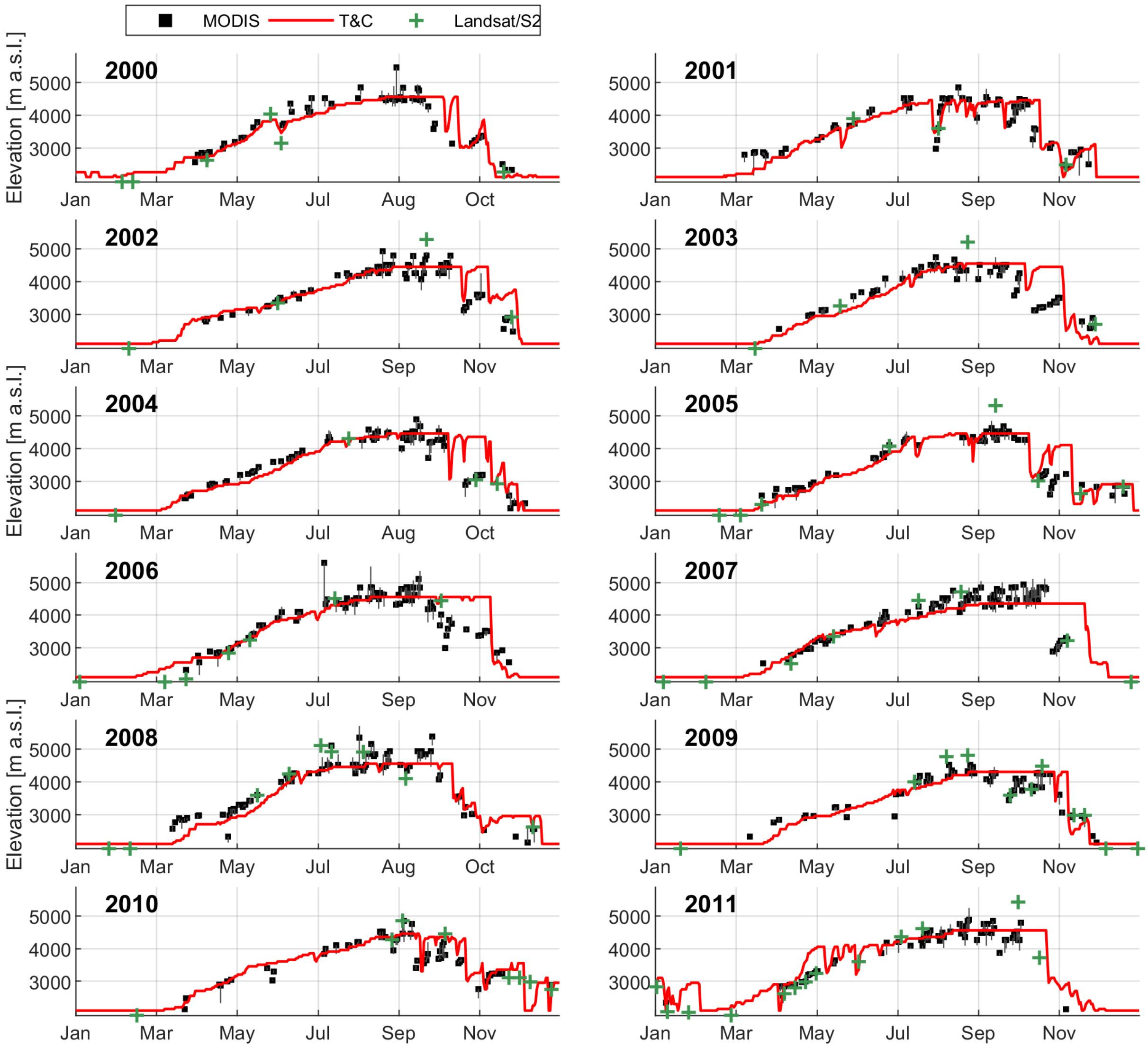

Figure S31. Evaluation of simulated snow line altitude against MODIS, Landsat 5/7/8/9, and Sentinel-2, from 2000 to 2012 (2012 to 2023 is shown in Fig. S32). Only scenes with fractional snow cover greater than 5% and MODIS scenes with less than 10% cloud cover are used in this comparison. Modelled snow line altitudes are shown on the daily scale. Vertical thin black bars above and below the black squares indicate the possible ranges of the MODIS snow line altitude obtained using different NDSI thresholds for the snow classification (0.25 - 0.40 - 0.45).

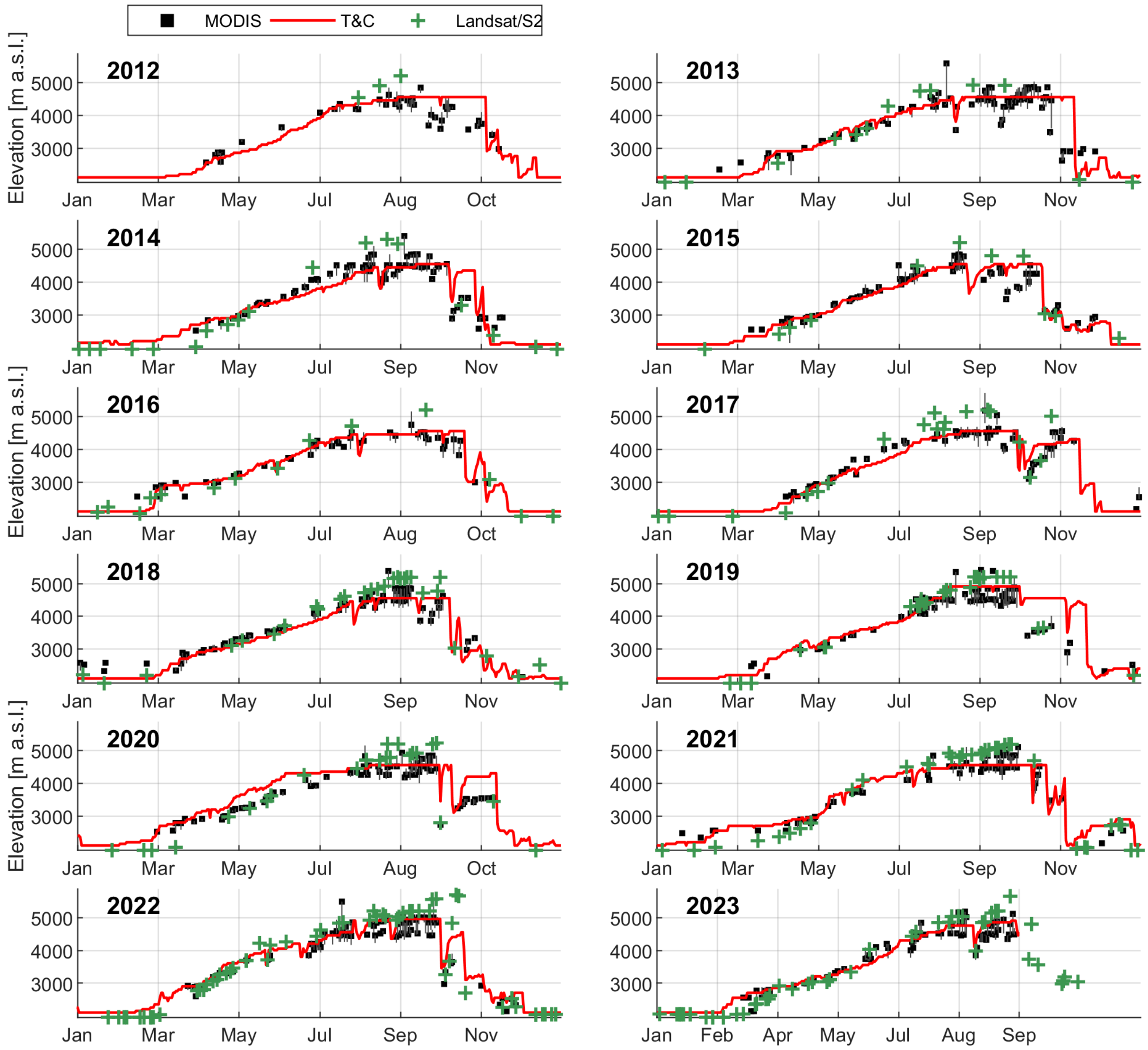

Figure S32. Evaluation of simulated snow line altitude against MODIS, Landsat 5/7/8/9, and Sentinel-2, from 2012 to 2023. Only scenes with fractional snow cover greater than 5% and MODIS scenes with less than 10% cloud cover are used in this comparison. Modeled snow line altitudes are shown on a daily scale. Vertical thin black bars above and below the black squares indicate the possible ranges of the MODIS snow line altitude obtained using different NDSI thresholds for the snow classification (0.25 - 0.40 - 0.45).

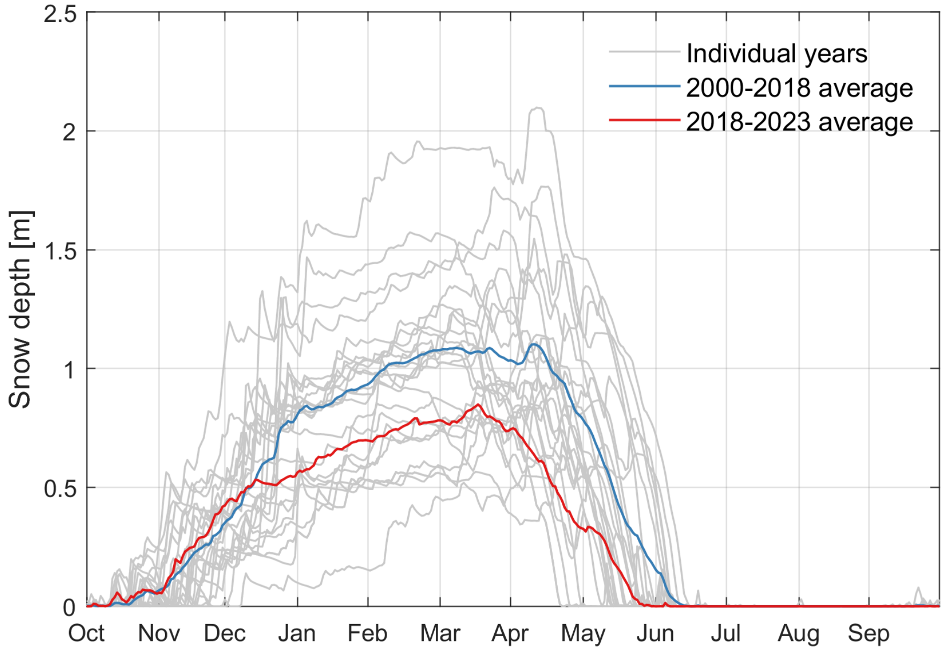

Figure S33. Snow height simulations at the pluviometer station, showing the average snow height for the two sub-periods and exhibiting a decrease in the snow cover duration during spring.

6. Supplementary Methods 6: Cut-off year for sub-periods comparison

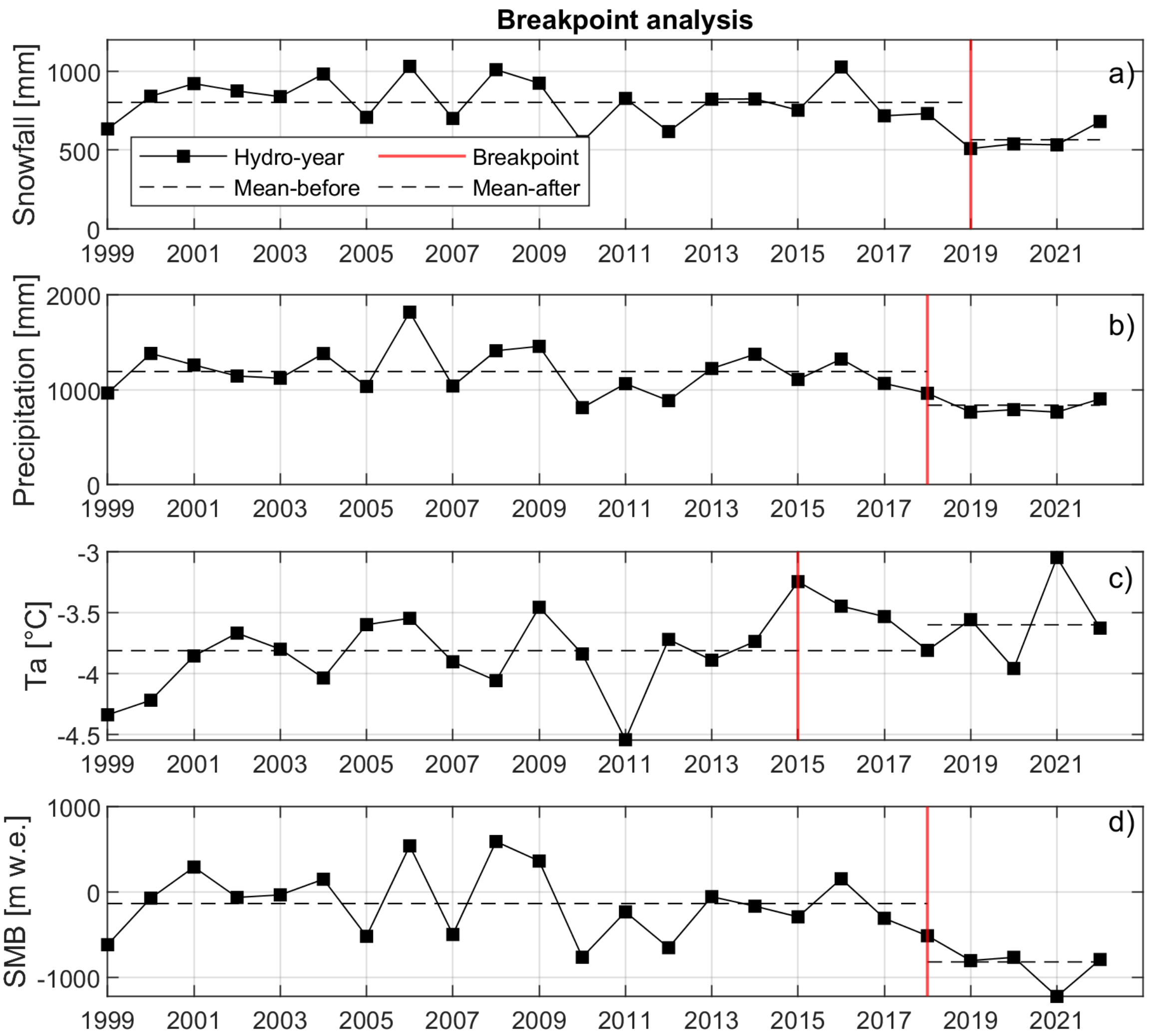

Figure S34: Breakpoint analysis conducted over four key variables using the *ischange* function of Matlab, which detects the point at which the mean changes the most. (a) Snowfall (b) Total precipitation. (c) Air temperature. (d) Glacier-wide surface mass balance. (a-c) variables are averaged over the catchment area, while the surface mass balance is for Kyzylsu Glacier. The simulated glacier mass balance for the period 2000-2012 is -0.06 m w.e./yr, while it is -0.22 +/- 0.27 m w.e./yr for the period 2012-2018 and -0.82 m w.e./yr for 2018-2023.

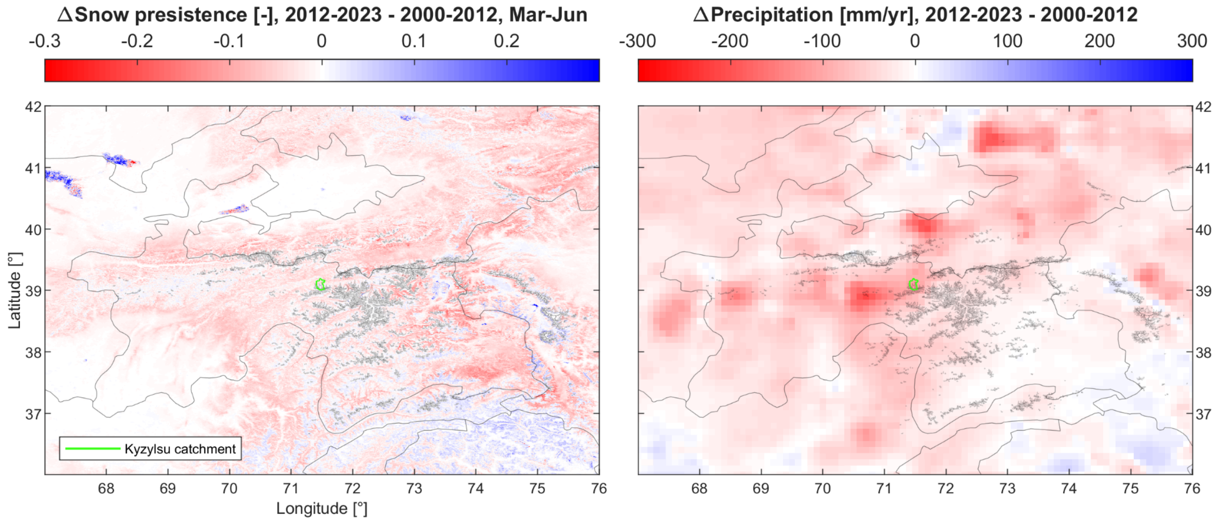

Figure S35. Regional anomalies in precipitation and snow cover, comparing 2000-2012 and 2012-2023. (a) Anomaly in mean snow cover persistence derived from the daily MODIS snow cover product (MOD10A1.061), focusing on the March to June period. (b) Regional anomaly in annual precipitation from ERA5-Land. World administrative boundaries (countries and territories) and glaciers from the RGI 6.0 inventory are shown as light grey solid lines. Our study catchment outlines are highlighted in green.

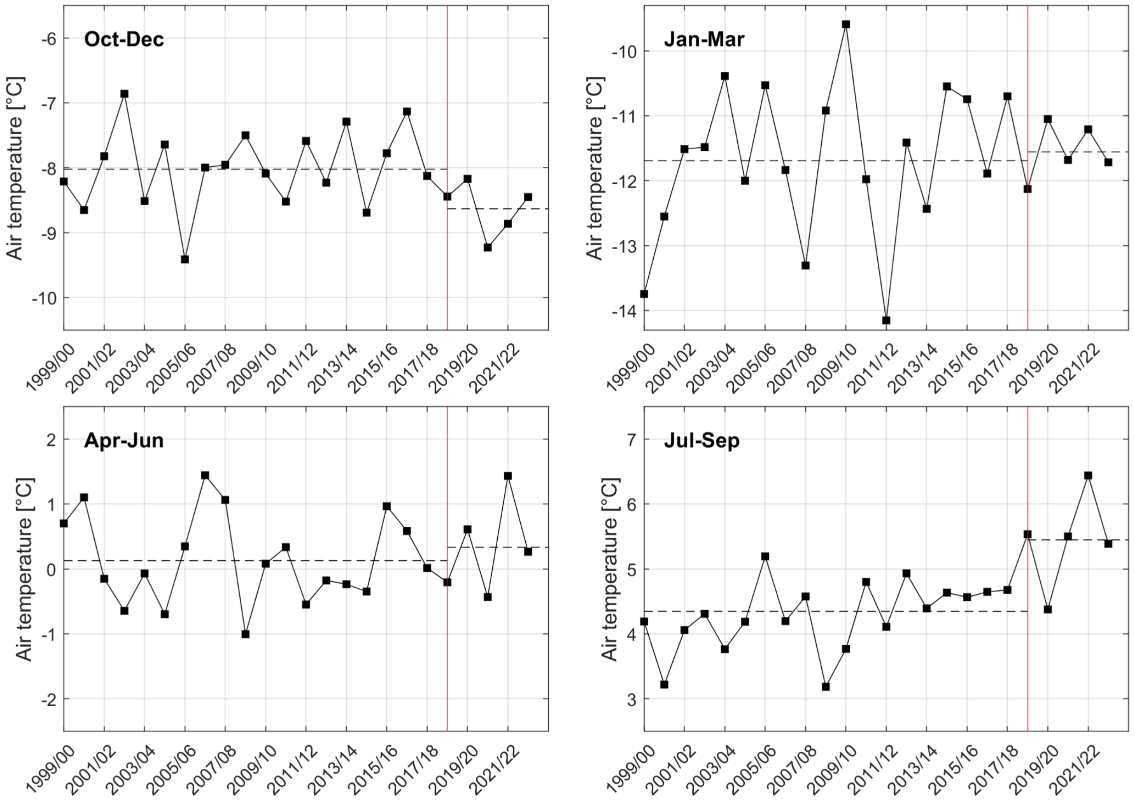

Figure S36. Mean seasonal air temperature averaged over the Kyzylsu catchment since October 1999. The horizontal dashed lines indicate the average over the period 1999-2018 and 2018-2023.

7. Supplementary Note 1: Seasonal and altitudinal additional model outputs

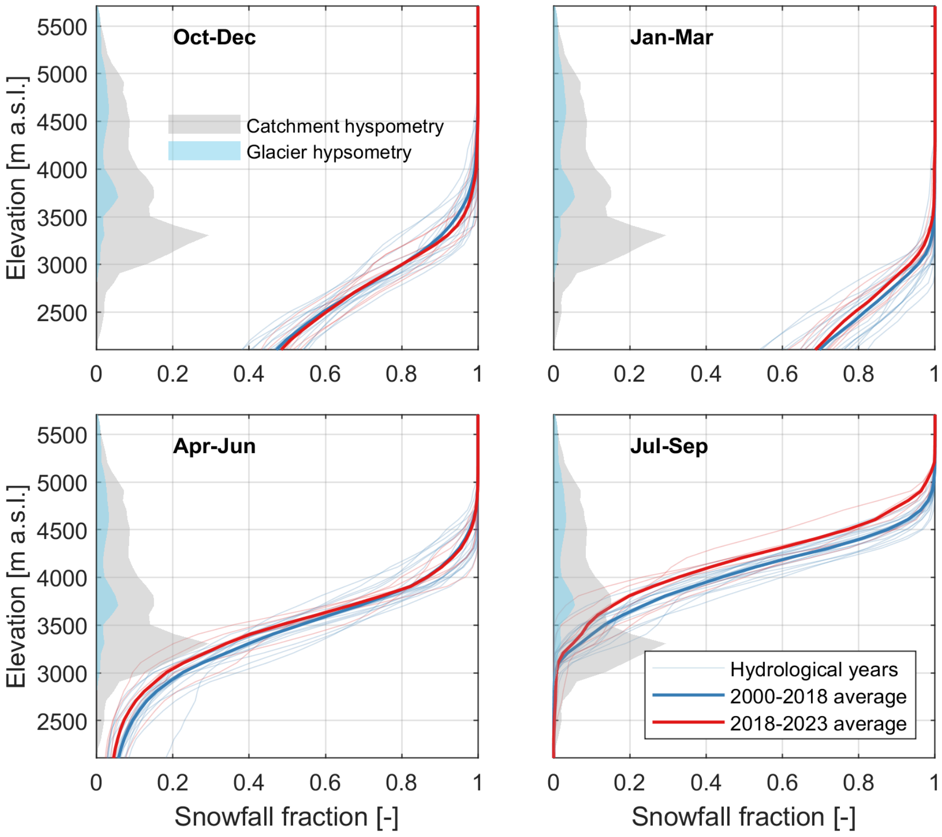

Figure S37. Mean seasonal snowfall fraction per 100-m elevation bands, for each individual year (thin lines) and averaged over the 2000-2018 and 2018-2023 periods.

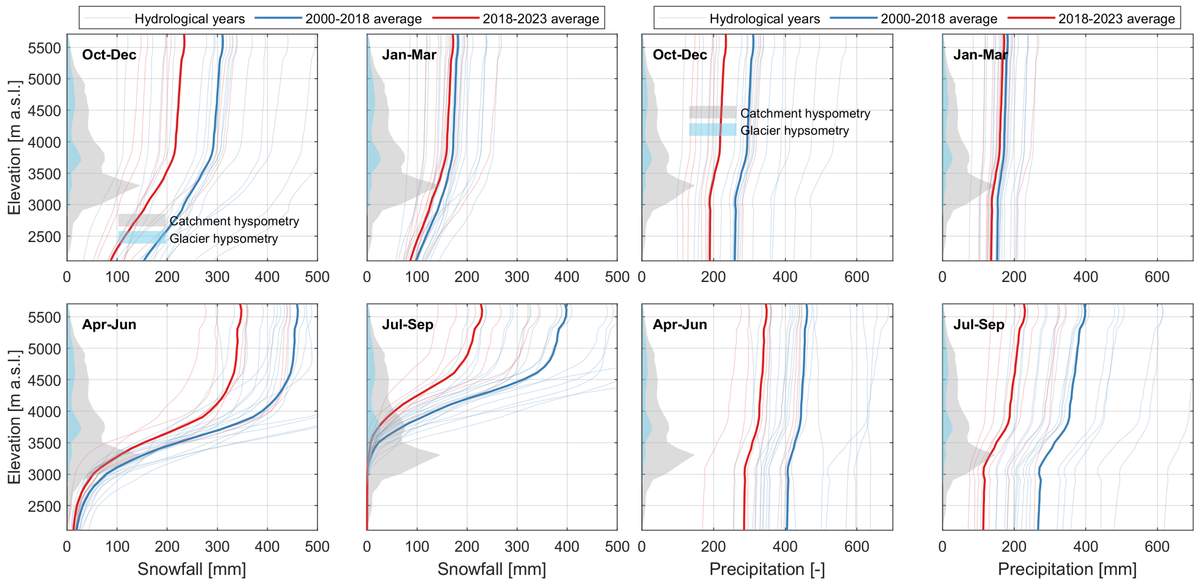

Figure S38. Mean seasonal snowfall and precipitation per 100-m elevation bands, for each individual year (thin lines) and averaged over the 2000-2018 and 2018-2023 periods.

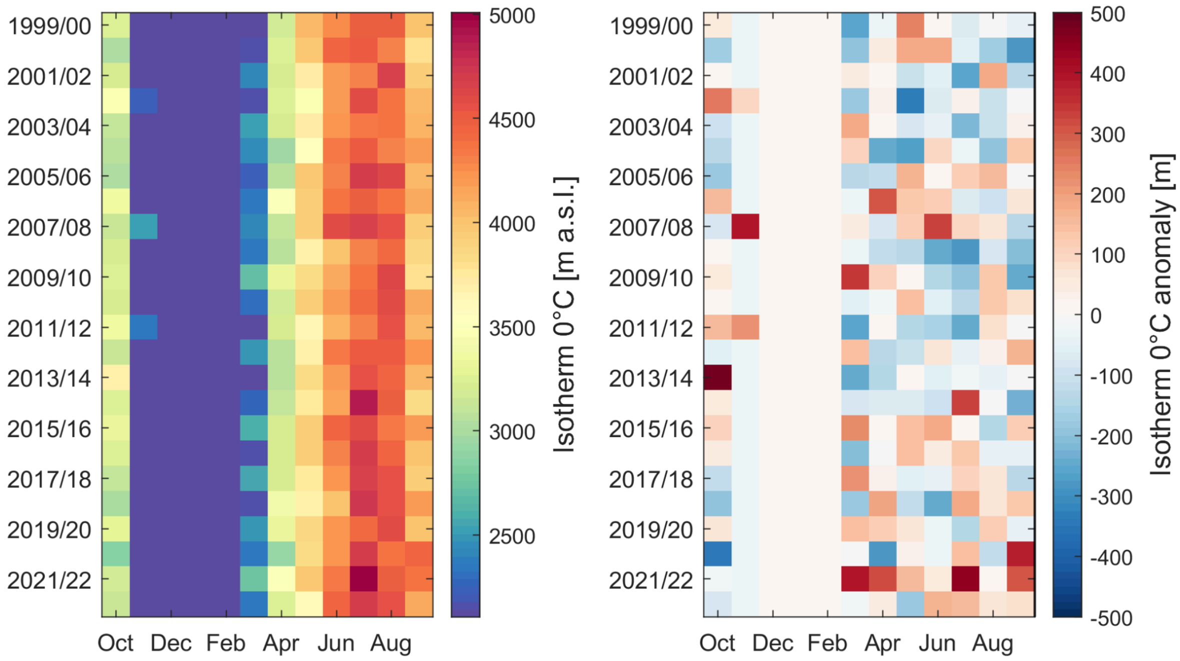

Figure S39. (a) Mean monthly elevation of the 0°C isotherm at the Kyzylsu catchment, from 1999 to 2023. (b) Mean monthly isotherm 0°C anomaly relative to the mean of all years in a given month.

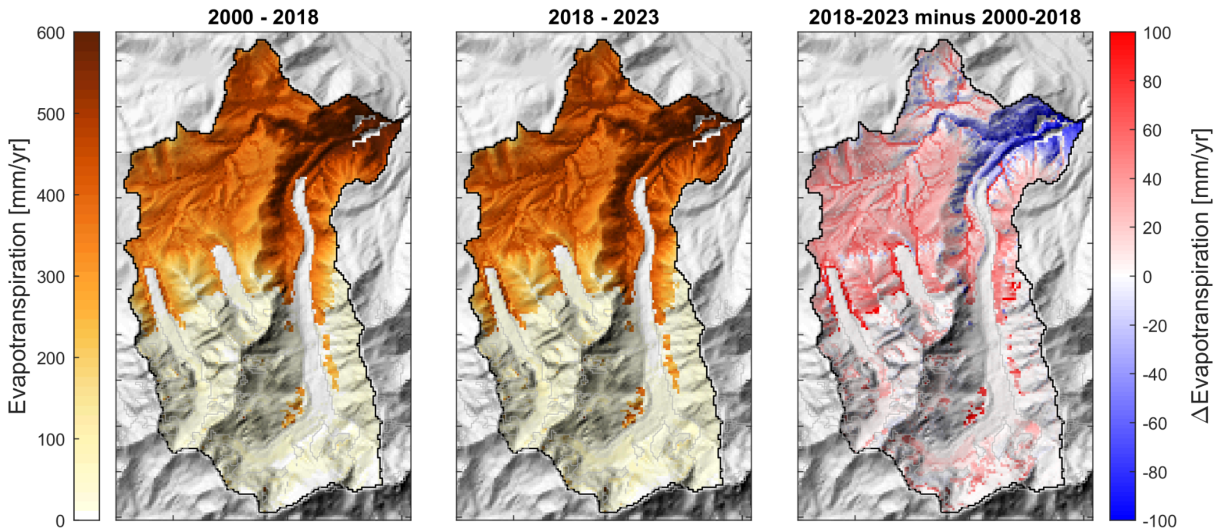

Figure S40. Mean annual evapotranspiration simulated by the land-surface model for the periods 2000-2018 (a), 2018-2023 (b) and shown as a difference between the two sub-periods (c). The hillshade derived from the AW3D surface DEM used for the model simulations is displayed in the background.

8. Supplementary Note 2: Linear regression analyses

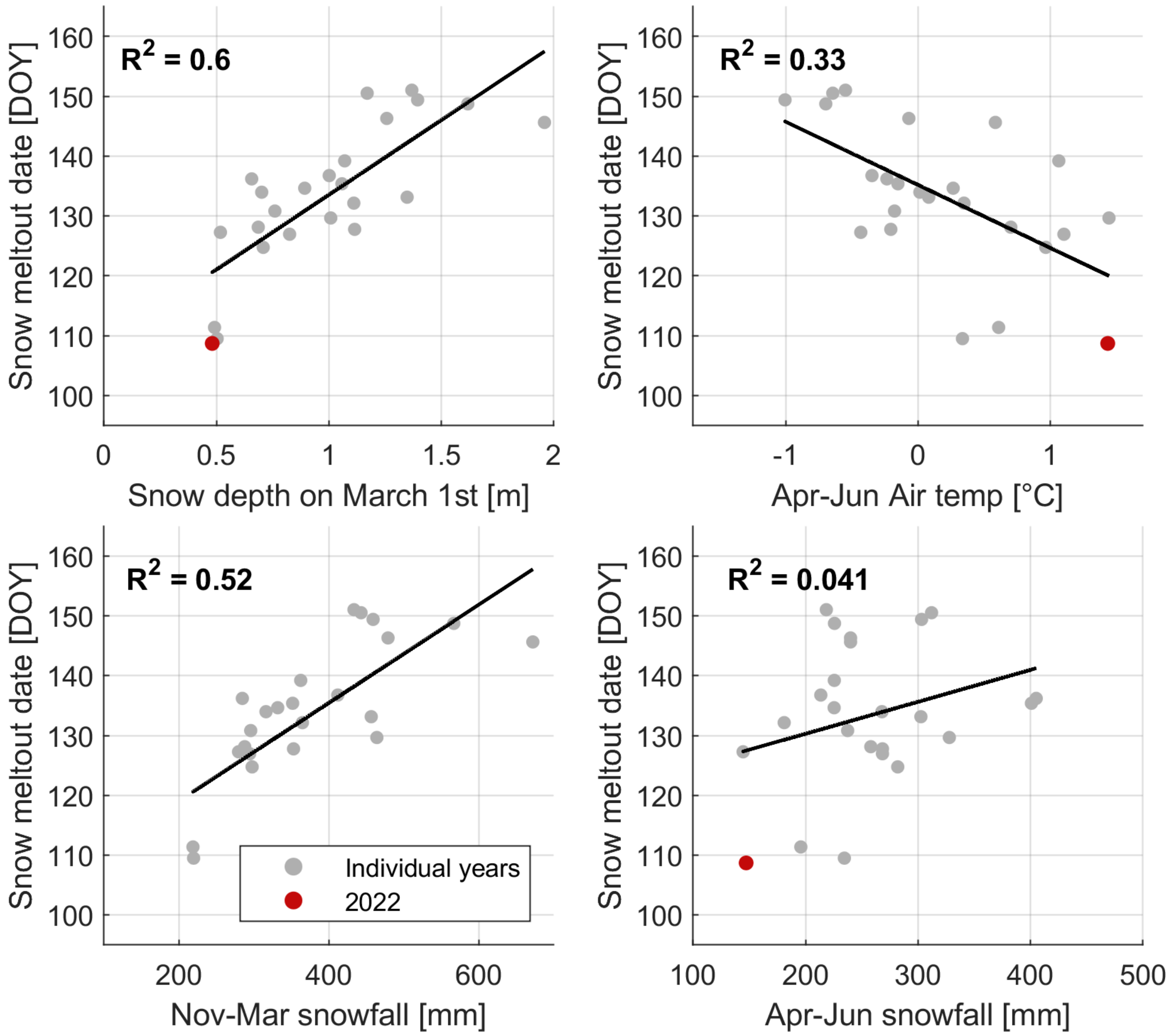

Figure S41. Linear regression between the yearly mean snowpack meltout date and the yearly mean snowpack height on March 1st (top-left), April-June mean air temperature (top-right), November-March snowfall (bottom-left), and April-June snowfall (bottom-right). The snowpack meltout date and snow depth were averaged over the catchment area located below 4000m a.s.l., elevation above which the snowpack does not always melt out in summer. The number given in the top-left corner of each panel is the coefficient of determination. Each dot represents a hydrological year.

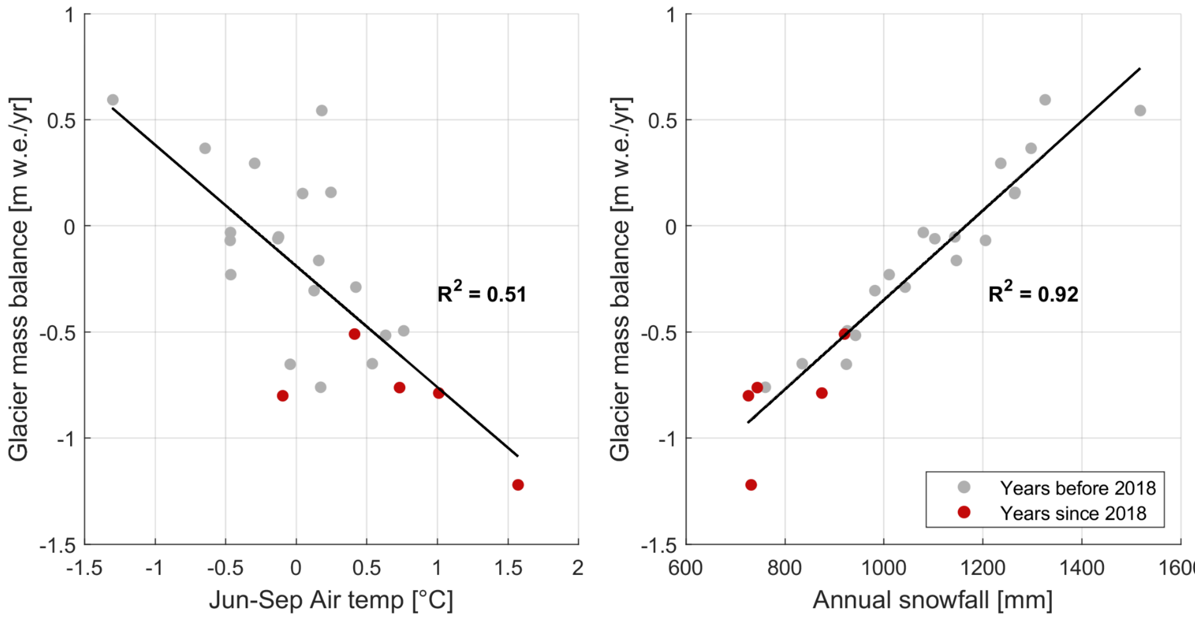

Figure S42. Linear regression between the simulated mass balance of Kyzylsu Glacier and the June-September mean air temperature (left) or annual snowfall (right). The number given on each panel is the coefficient of determination. Each dot represents a hydrological year.

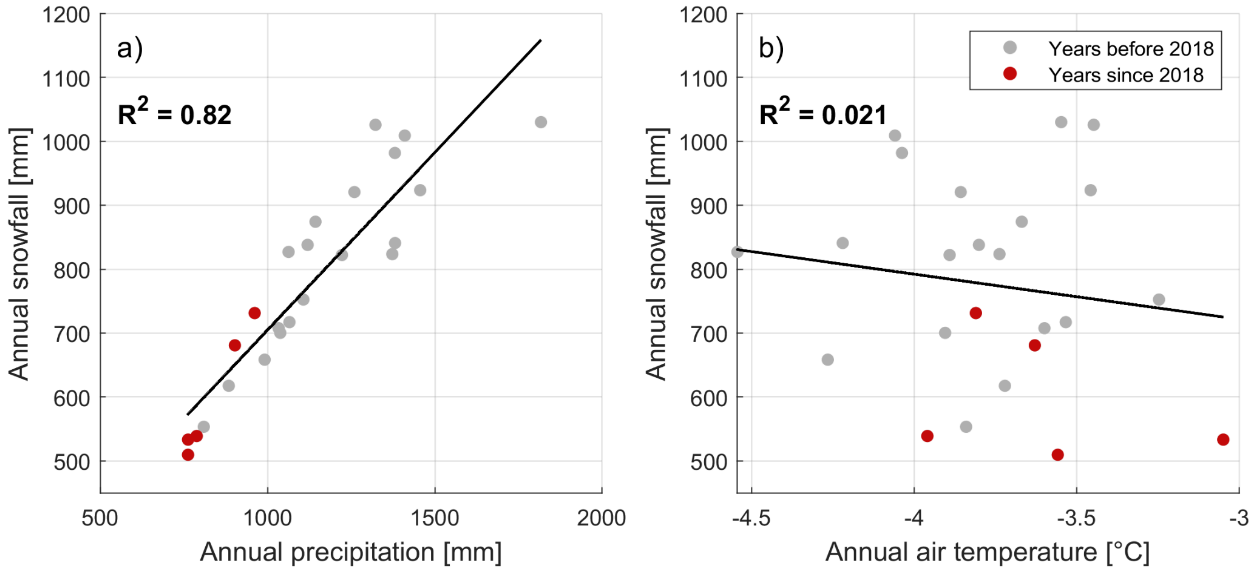

Figure S43: Linear regression between the annual snowfall and annual precipitation (a), and between annual snowfall and mean annual air temperature (b). These variables correspond to catchment-wide averages. The number given in the top-left corner of each panel is the coefficient of determination. Each dot represents a hydrological year.

9. Supplementary Note 3: Additional material

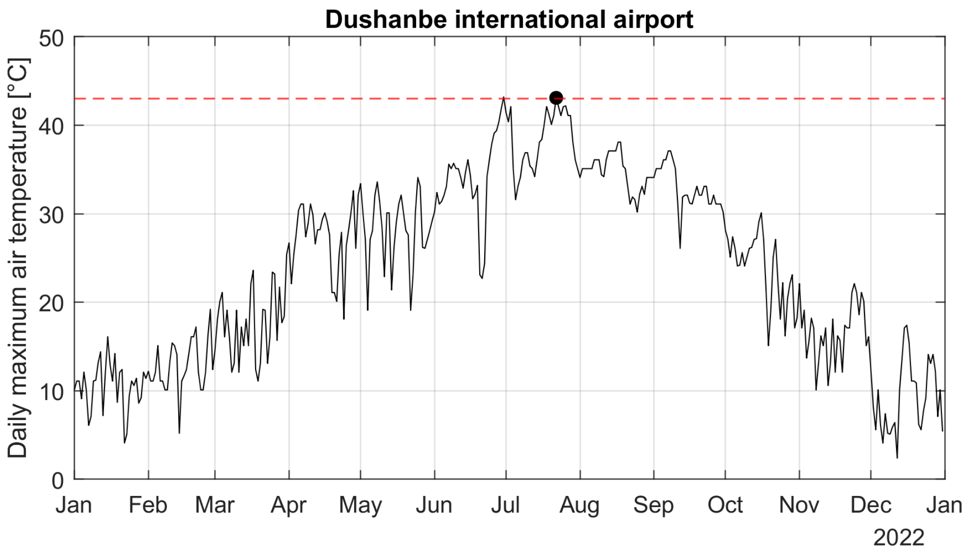

Figure S44. Daily maximum air temperature recorded in 2022 at the weather station located at the Dushanbe International Airport (700 m a.s.l.). The red dashed line indicated the 43°C degree, which was reached on 22 July 2022, as indicated by the black dot. The data was retrieved from the following webpage: https://www.visualcrossing.com/weather-history/38836099999/metric/2022-01-01/2022-12-31

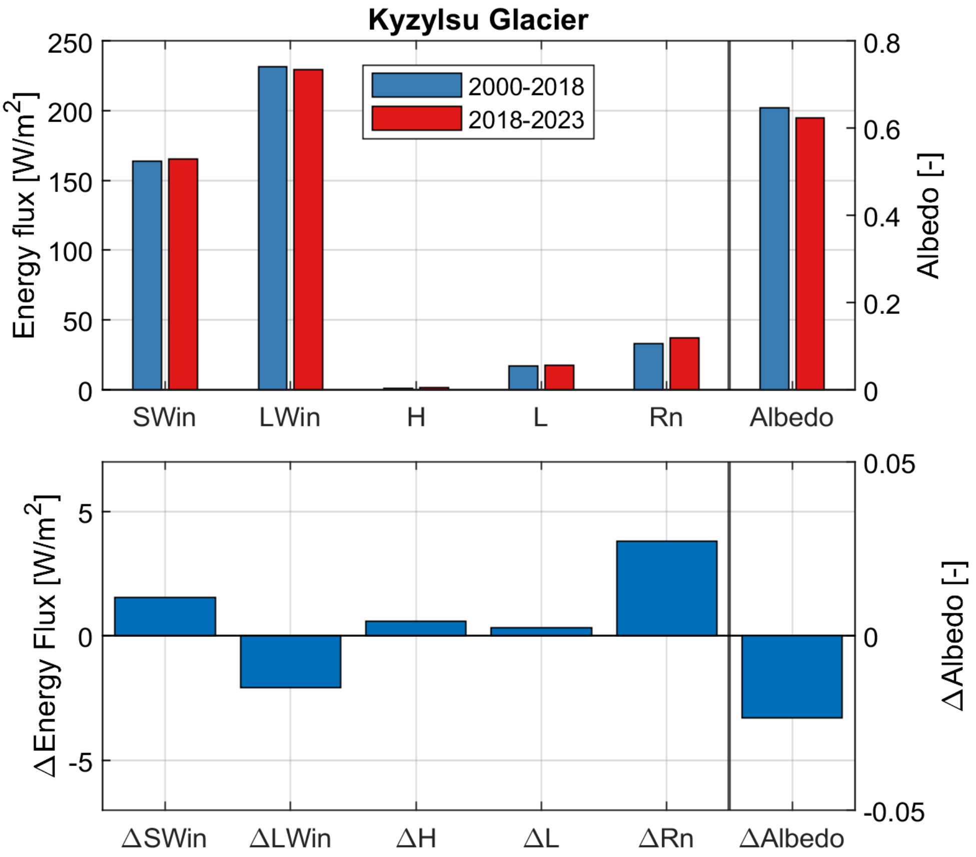

Figure S45. Energy fluxes and surface albedo of Kyzylsu Glacier, for the periods 2000-2018 and 2018-2023 (top panel), and their differences (bottom panel). These fluxes were

averaged over the whole area of Kyzylsu Glacier. The variables are, from left to right: SWin = incoming shortwave radiation, LWin = incoming longwave radiation, H = sensible heat flux, L = latent heat flux, Rn = net radiation, Albedo = surface albedo.

10. Supplementary References

1. Farinotti, D. et al. A consensus estimate for the ice thickness distribution of all glaciers on Earth. Nature Geoscience 12, 168–173 (2019).

2. Kneib, M. et al. Controls on Ice Cliff Distribution and Characteristics on Debris-Covered Glaciers. Geophysical Research Letters 50, e2022GL102444 (2023).

3. Goerlich, F., Bolch, T. & Paul, F. More dynamic than expected: An updated survey of surging glaciers in the Pamir. Earth System Science Data 12, 3161–3176 (2020).

4. Hugonnet, R. et al. Accelerated global glacier mass loss in the early twenty-first century. Nature 592, 726–731 (2021).

5. Kronenberg, M. et al. Long-term firn and mass balance modelling for Abramov Glacier in the data-scarce Pamir Alay. Cryosphere 16, 5001–5022 (2022).
